# Short-term Preoperative Dietary Restriction Is Neuroprotective in a Rat Focal Stroke Model

**DOI:** 10.1371/journal.pone.0093911

**Published:** 2014-04-04

**Authors:** Kärt Varendi, Mikko Airavaara, Jenni Anttila, Sarah Vose, Anu Planken, Mart Saarma, James R. Mitchell, Jaan-Olle Andressoo

**Affiliations:** 1 Institute of Biotechnology, University of Helsinki, Helsinki, Finland; 2 Department of Genetics and Complex Diseases, Harvard School of Public Health, Boston, Massachusetts, United States of America; University of South Florida, United States of America

## Abstract

Stroke is a major complication of cardiovascular surgery, resulting in over 100,000 deaths and over a million postoperative encephalopathies annually in the US and Europe. While mitigating damage from stroke after it occurs has proven elusive, opportunities to reduce the incidence and/or severity of stroke prior to surgery in at-risk individuals remain largely unexplored. We tested the potential of short-term preoperative dietary restriction to provide neuroprotection in rat models of focal stroke. Rats were preconditioned with either three days of water-only fasting or six days of a protein free diet prior to induction of transient middle cerebral artery occlusion using two different methods, resulting in either a severe focal stroke to forebrain and midbrain, or a mild focal stroke localized to cortex only. Infarct volume, functional recovery and molecular markers of damage and protection were assessed up to two weeks after reperfusion. Preoperative fasting for 3 days reduced infarct volume after severe focal stroke. Neuroprotection was associated with modulation of innate immunity, including elevation of circulating neutrophil chemoattractant C-X-C motif ligand 1 prior to ischemia and suppression of striatal pro-inflammatory markers including tumor necrosis factor α, its receptor and downstream effector intercellular adhesion molecule-1 after reperfusion. Similarly, preoperative dietary protein restriction for 6 days reduced ischemic injury and improved functional recovery in a milder cortical infarction model. Our results suggest that short-term dietary restriction regimens may provide simple and translatable approaches to reduce perioperative stroke severity in high-risk elective vascular surgery.

## Introduction

Perioperative stroke occurring during or soon after surgery is a major cause of morbidity and mortality, with an average incidence of 2–13% in cardiovascular procedures and 0.08–0.7% in non-cardiovascular procedures [1], [2]. With 7 million cardiovascular and 21 million non-cardiovascular surgeries performed annually in the US [3] and similar numbers in Europe (extrapolated from data from the Netherlands [4]), annual deaths are calculated to be in excess of 180,000 on these two continents alone. Those left with the debilitating consequences of perioperative stroke/encephalopathy number an order of magnitude higher [5], [6]. Treatment of perioperative stroke accounts for a quarter of the resources spent annually for stroke treatment in the USA [5].

Numerous pharmacological compounds have been tested for their ability to provide neuroprotection after stroke, including 5-HT_1a_ agonists, free radical scavengers, immunosuppressants and agents that block excitotoxicity. Despite efficacy in rodent models, most have failed in clinical trials [7]. While perioperative stroke risk assessment prior to surgery is a common practice, general prophylactic methods are lacking [1], [2], underpinning a need for basic research.

Strategies that provide neuroprotection when initiated before the ischemic period are known as preconditioning. Ischemic preconditioning is a phenomenon in which brief periods of ischemia protect against subsequent, longer insults to various organs, including heart [8] and brain [9]. In preclinical models of stroke, ischemic preconditioning prevents subsequent ischemic injury by suppressing the expression of pro-inflammatory cytokines, chemokines, adhesion molecules and transcription factors [10]. Other low-dose stressors such as hypoxia [11], endotoxin [12] or heat shock [13] can also precondition against ischemic injury. However, the clinical application of such methods has remained a matter of debate in large part due to the potential safety concerns, highlighting a need for safer preoperative prophylactic methods.

Dietary restriction (DR), defined as reduced food intake without malnutrition, extends lifespan and increases resistance to a variety of acute stressors in multiple species, including rodents [14]. Long-term application of DR for 3 months or longer is neuroprotective in rodent models of stroke [15] and excitotoxicity [16]–[18]. Mechanistically, upregulation of neurotrophic and growth factors, such as brain-derived neurotrophic factor (BDNF) induced by long-term DR [19], [20] could be partially responsible for increased protection, as BDNF has been shown to reduce neuronal injury after ischemia [21], [22]. Long-term DR also offers benefits against ischemic injury in other organs, such as heart, through a variety of mechanisms including immunosuppression, elevation of reactive oxygen and nitrogen species scavenging mechanisms and upregulation of heat shock protein levels [23], [24].

Despite its potential as a safe and effective prophylactic method, the relatively long periods of food restriction employed in preclinical studies (3 months or longer) are not considered feasible in a clinical setting [25]. However, recent data indicate that dietary preconditioning against ischemic injury can be realized in a clinically relevant time frame in rodent models [26]. For example, 2 weeks of 30% reduced daily food intake or 3 days of water-only fasting protect against ischemia reperfusion injury to kidney or liver [27]. Protein restriction in the absence of calorie restriction, or restriction of individual essential amino acids such as tryptophan, can also impart benefits within 6 days [28]. Here, we describe two different pre-operative manipulations – 3 days of water-only fasting and 6 days of protein-free DR – with benefits on focal stroke outcomes in rats.

## Methods

Please also see Methods S1 for further details.

### Animals

All animal experiments were carried out according to the National Institute of Health (NIH) guidelines for the care and use of laboratory animals and approved by the appropriate local or national ethics board (permit number ESAVI/5459/04.10.03/2011, issued by ELÄINKOELAUTAKUNTA – ELLA Etelä-Suomen aluehallintovirasto, Finland). Adult male Sprague-Dawley (SD) rats weighing 240–300 g were housed under standard conditions with *ad libitum* access to food and water unless indicated otherwise.

### Dietary preconditioning regimens

Fasting was performed by removing the complete chow diet (Harlan Teklad Global 2016 Rodent Diet) for 3d while maintaining free access to water at all times. Protein-free dietary restriction was performed by first acclimating all animals to a complete diet made of refined ingredients (Research Diets D12450B) consisting of 18% calories from protein (casein), 72% from carbohydrate (corn starch, maltodextrin, sucrose) and 10% from fat (soybean oil, lard) for 6d. The control group was then maintained on the complete diet, and the protein-free group was given restricted access to an isocaloric diet lacking protein (Research Diets D08043003, consisting of 90% calories from carbohydrate and 10% from fat) at 60% of the average daily intake of the complete diet group for 6d prior to tMCAO and 2d after reperfusion.

### Surgical procedures

Two different stroke models were employed. In experiments testing the effects of fasting, a severe focal stroke involving forebrain and midbrain was induced by intraluminal occlusion of the middle cerebral artery (MCA) with a filament for 60 min, followed by reperfusion as described previously [29]. Stroke involving forebrain and midbrain is associated with fever [30]. To determine whether the MCA occlusion surgery produced a lesion, core body temperature in each animal one hour after reperfusion was measured. Experiments evaluating the effect of fasting on severe focal stroke were performed at Charles River Laboratories (CRL), Kuopio, Finland.

In experiments testing the effects of protein-free DR, a mild focal stroke involving cortex only was induced by transient direct occlusion of the right MCA and bilateral CCAs with a 10-0 suture for 60 min followed by reperfusion as described previously [31]. Cortical-only stroke does not result in fever in rats [32] (Figure S1) thus the presence of lesion was verified by behavioral tests up to two weeks post-stroke and/or by TTC staining 48 hours after stroke. Experiments evaluating the effect of protein-free diet on “mild” cortical stroke were performed at the University of Helsinki (UH), Finland.

### Behavioral procedures

In the mild suture-induced cortical stroke model, neurological deficits were evaluated using body swing, Bederson's score [33] and cylinder tests [31]; and locomotor activity was measured using an infrared activity monitor (MedAssociates Inc.). All tests were conducted by an investigator blinded to the treatment groups.

### Analysis of infarction volume

For determination of infarct volume 7d after severe filament-induced stroke, T2-weighted multi-slice (12–14 continuous slices) MRI images were acquired using a Varian Inova console interfaced to a 4.7T horizontal magnet (Magnex Scientific Ltd, Abington, UK). Lesion quantitation was done by manually delineating total lesion outlines from MRI images based on T2 contrast between lesioned and healthy tissue using MATLAB software by an observer blinded to the treatment groups.

Infarct volume 2d after mild suture-induced cortical stroke was assessed with triphenyltetrazolium chloride (TTC) staining by an observer blinded to the treatment groups as described previously [31].

### Blood measurements

Glucose levels were measured from fresh blood with a Glucocard II Super device (Akray Factory Inc., Shiga, Japan). Plasma cytokines were measured on the Rat Demonstration Multi-Spot plate (Meso Scale Discovery, Gaithersburg, MD) according to the manufacturer's instructions. Clinical chemistry analyses from plasma samples were performed with an automatic analyzer (Konelab 30i, Thermo Fisher Scientific, Vantaa, Finland) according to manufacturer's instructions.

### Real-time quantitative PCR

Striatum was dissected from snap frozen brains before or 24hr after severe filament-induced stroke for isolation of RNA for cDNA synthesis. Real-time quantitative PCR (qPCR) was performed on a Lightcycler®480 real-time PCR system (Roche Diagnostics) using Lightcycler®480 SYBR Green I Master complemented with 2.5pmol of primers (Table S1). Reactions were performed in triplicate and analyzed with Lightcycler®480 Software. Gene expression was normalized to peptidylprolyl isomerase A. Similar methods were employed for gene expression analysis from cortex and striatum before or 24hr after mild suture-induced stroke.

### Statistics

All values are presented as mean ± standard error of the mean (SEM). Differences were considered to be statistically significant at the p<0.05 level. Statistical analyses including Student's t-test, Mann-Whitney *U*-test and one- or two-way ANOVA or Kruskal-Wallis non-parametric ANOVA followed by appropriate *post hoc* analysis were performed with SPSS 15.0 software.

## Results

### Neuroprotection against severe focal stroke by preoperative fasting

**Figure 1:**
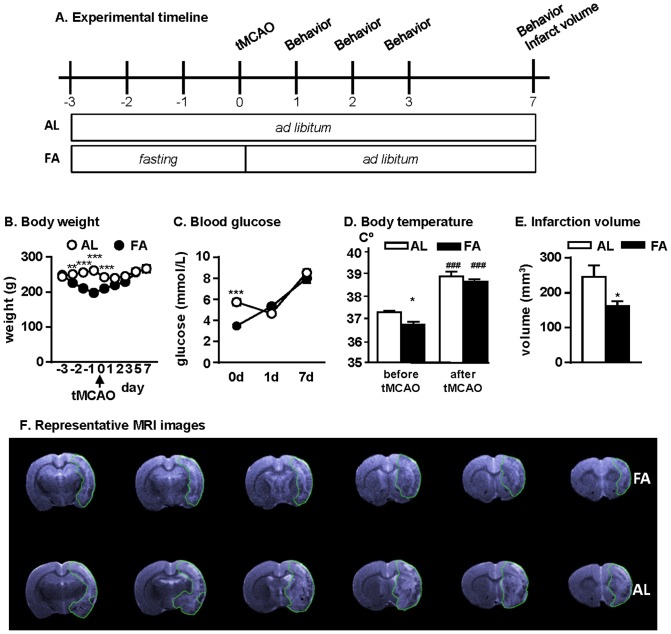
Preoperative 3-day water-only fasting is neuroprotective against stroke. (A) Experimental timeline indicating periods of *ad libitum* feeding and fasting relative to the onset of tMCAO on day 0. AL, *ad libitum* fed (n = 11); FA, fasted (n = 14). (B) Average body weights prior to and after tMCAO; F_8, 23_ = 17.69, ***p<0.0001, 2-way ANOVA. (C) Blood glucose levels on the indicated days prior to and after tMCAO; F_2, 23_ = 14.09, ***p<0.0001, 2-way ANOVA. (D) Body temperature before tMCAO and 1 hour after reperfusion; ***/^###^p<0.001, Student's *t*-test. (E) Infarction volumes at d7 after tMCAO; *p = 0.0215, Student's *t*-test. (F) Representative MRI images of the lesioned brain sections with green lines surrounding the lesion.

In hypoxia and global brain ischemia models, short-term water-only fasting is protective [34]–[38]. However, while global brain hypoperfusion accounts for less than 10% of perioperative strokes in humans [2], the majority (62%) of perioperative strokes are caused by focal ischemic insults that are mechanistically and pathologically different from hypoperfusion. Our first objective was to assess the effect of short-term fasting on severe focal brain ischemia. Rats were subjected to pre-operative water-only fasting for 3d as shown in Fig 1A. As expected, fasted rats exhibited a significant reduction in body weight (Fig 1B). Fasting also significantly reduced blood glucose levels (Fig 1C) and body temperature (Fig 1D) prior to induction of focal stroke by intraluminal occlusion of the middle cerebral artery (MCA) with a filament for 60 min. One hour after reperfusion, body temperature was elevated in both groups, consistent with lesion induction (Fig 1D). Analysis of infarction volumes obtained from T2-MRI images revealed a significant reduction 7d after tMCAO in the fasted group relative to the *ad libitum*-fed group (Fig 1E and F).

### Suppression of pro-inflammatory response to severe focal stroke by fasting

**Figure 2:**
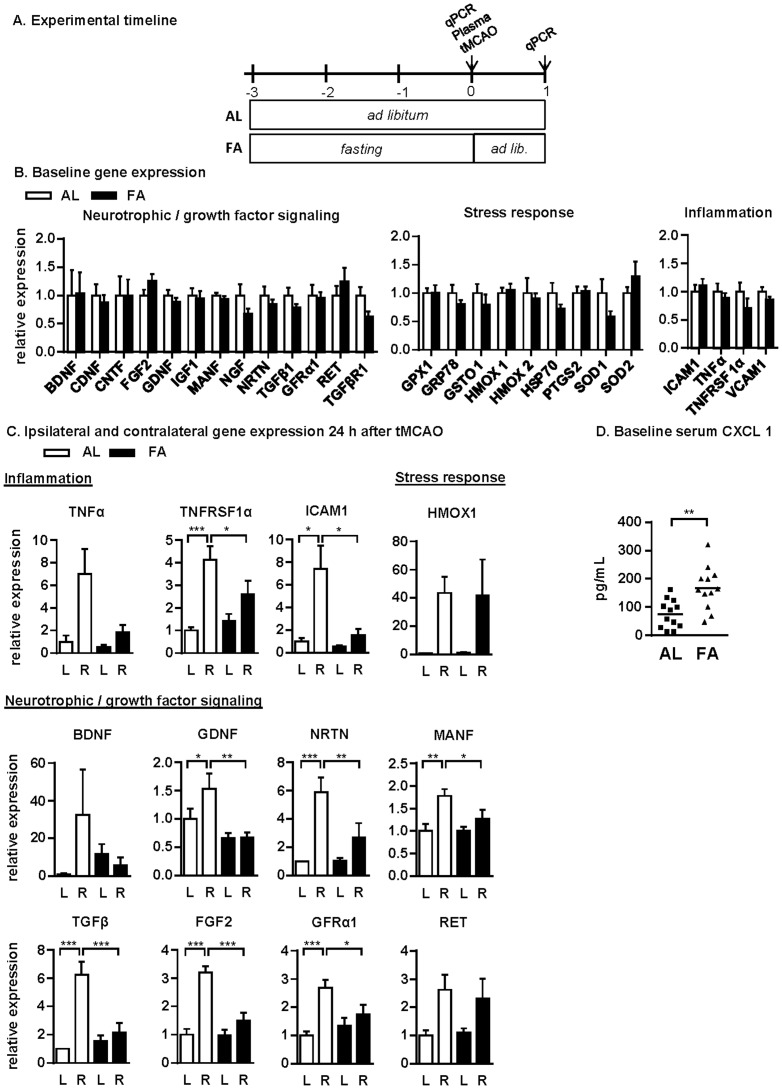
Molecular mechanisms of fasting-induced neuroprotection. (A) Experimental timeline indicating dietary treatments and experimental endpoints relative to tMCAO on day 0. (B) Relative expression of the indicated genes in the striatum of *ad libitum* fed (AL, n = 6) and fasted (FA, n = 5) rats at baseline, measured by qPCR and expressed relative to the AL group. (C) Relative expression of selected genes in AL (n = 5) and FA (n = 6) rats 24 hours after tMCAO in the unlesioned left (L) and lesioned right (R) striata, measured by qPCR and expressed relative to the unlesioned AL group; *p<0.05, **p<0.01, ***p<0.001, 1-way ANOVA. (D) Serum CXCL1 levels in AL (n = 12) and FA (n = 12) rats at baseline; **p = 0.0021, Student's *t*-test.

Long-term DR is believed to protect against ischemia reperfusion injury at least in part through suppression of inflammatory responses [23]. In the context of neuroprotection, long-term DR is additionally associated with upregulation of neurotrophic factors and growth factors such as BDNF [16], [17], [19], [20], and increased expression of proteins involved in cytoprotection [39]. To gain insight into the molecular mechanisms of protection by fasting against focal stroke, we analyzed striatal gene expression immediately before (baseline) and 24hr after tMCAO using qPCR (Fig 2A). Unlike long-term DR, we found no significant differences in mRNA expression levels of growth factor-related, cytoprotective or pro-inflammatory markers at baseline as a result of 3d of fasting relative to *ad libitum* (AL) fed controls (Fig 2B, Table S1). 24hr after reperfusion, expression of mRNAs encoding for the pro-inflammatory cytokine tumor necrosis factor alpha (TNFα), its receptor TNFRSF1A and its downstream target, the intercellular adhesion molecule 1 (ICAM1) were significantly upregulated in the lesioned hemisphere compared to the control hemisphere in the *ad libitum* group, but not in the fasted group (Fig 2C), suggesting attenuation of the inflammatory response to stroke in fasted rats. Surprisingly, mRNAs encoding for several neurotrophic and growth factors including glial cell line-derived neurotrophic factor (GDNF), neurturin (NRTN), mesencephalic astrocyte-derived neurotrophic factor (MANF), transforming growth factor beta 1 (TGFβ1), fibroblast growth factor 2 (FGF2) and GDNF family receptor alpha 1 (GFRα1) were significantly upregulated in the lesioned striatum after stroke in the *ad libitum* rats but not in the fasted rats (Fig 2C). A similar trend was observed in BDNF mRNA expression, but this effect did not reach statistical significance. Interestingly, no significant differences were observed in the expression of cellular stress response genes (with the exception of HMOX1, Fig 2C) as a function of diet or stroke. The expression of all genes analyzed relative to the reference gene PPIA and normalized to expression in the *ad libitum* group at baseline is provided in Table S1; no significant changes were observed in any of these genes in sham-operated animals 24hr after operation in either dietary group.

Since DR can modulate innate immune activation, we measured the concentration of plasma chemokines/cytokines (CXCL1, IL-1b, IL-4, IL-5, TNFα, IFNγ and IL-13) prior to and 4hrs after tMCAO. Of these, only the pro-inflammatory neutrophil chemoattractant CXCL1 (C-X-C motif ligand 1) was significantly differentially regulated by fasting at baseline, with higher levels in the fasted group than in the *ad libitum* fed group (Fig 2D). Four hours after tMCAO, there was a trend in each of the seven cytokines tested toward being reduced in the pre-fasted group, but none reached statistical significance (Table 1).

**Table 1:** Plasma cytokine levels (pg/mL) 4 hours after reperfusion in rats fed *ad libitum* (AL) or fasted for 3 days (FA) prior to tMCAO; IFN, interferon; IL, interleukin, CXCL1, C-X-C motif ligand 1; TNF, tumor necrosis factor.

4hr plasma cytokines	IFNα	IL1β	CXCL1	TNFα	IL4	IL5	IL13
AL (n = 6)	11.7±3.8	49.3±9.7	9874±2069	25.4±6.3	7.2±1.7	132.7±26.1	0.3±0.3
FA (n = 6)	5.0±2.1	55.3±14.3	7205±2388	12.9±7.0	5.3±1.5	87.2±23.3	0.3±0.2
p-value	0.16	0.74	0.42	0.22	0.42	0.22	0.94

### Neuroprotection against mild focal cortical stroke by protein-free dietary restriction

Because water-only fasting may in some clinical settings be difficult to tolerate, we next asked whether a milder short-term food restriction regimen could also precondition against stroke. A protein-free DR preconditioning regimen was chosen based on its efficacy in protecting kidney and liver from ischemia reperfusion injury [28].

The intraluminal filament-induced severe stroke model used in the fasting experiments above is one of the most widely used focal stroke models in rats. However, a potential limitation of this method is the severity of the resulting infarction, since stroke lesion of comparable size extending from forebrain to midbrain in humans is most often fatal. We thus continued to probe the potential benefits of protein-free DR using a stroke model that causes a milder lesion restricted to cortex [40], which is a common type of embolic stroke in humans. Our objective was also to assess infarct size at its maximum, i.e. 48 h after stroke and evaluate functional outcome at 2, 7 and 14 days post-stroke.

**Figure 3:**
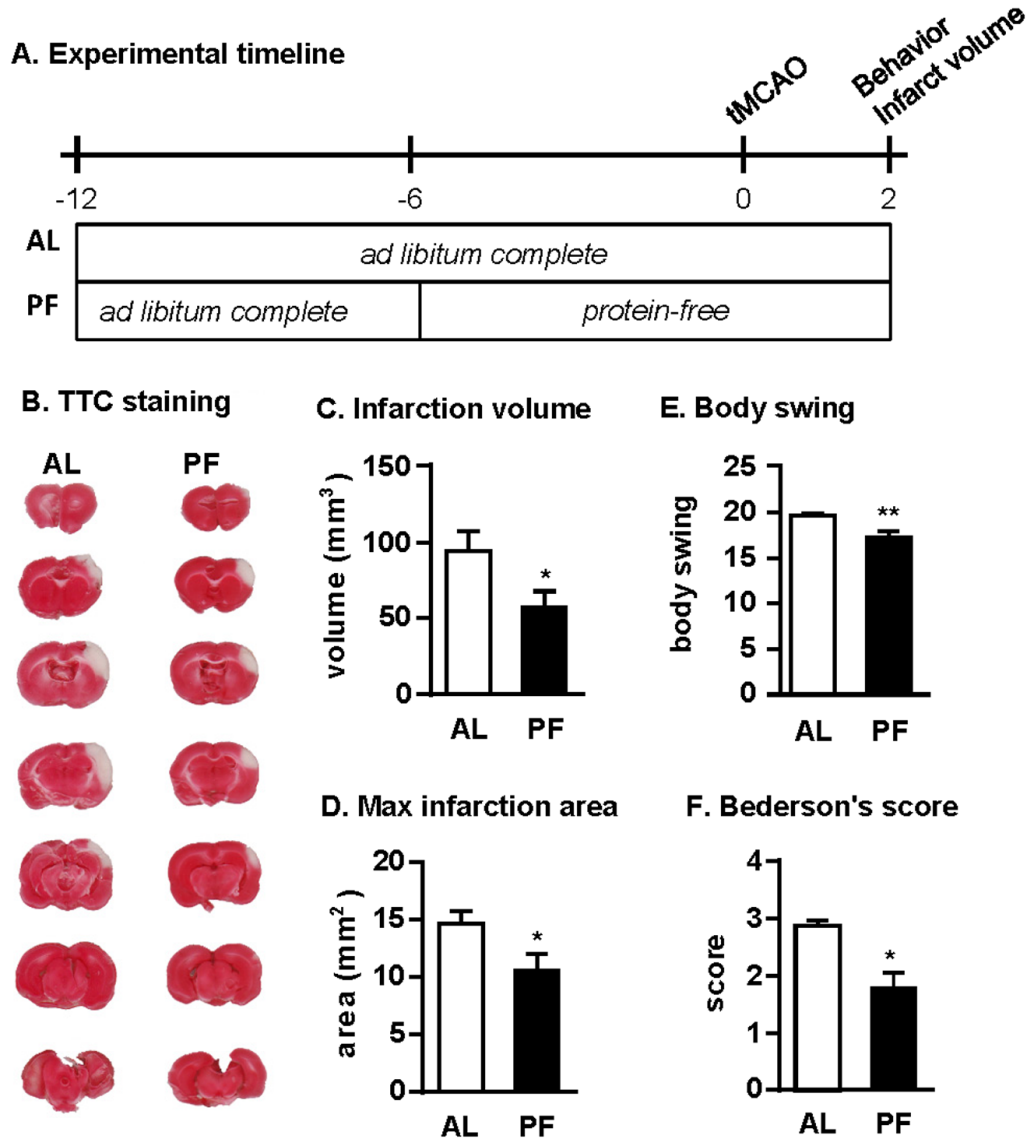
Protein-free DR is neuroprotective against stroke. (A) Experimental timeline indicating periods of *ad libitum* access to a complete diet (AL, n = 15) or restricted access to a protein-free diet (PF, n = 14) relative to the onset of tMCAO on day 0. (B) TTC-stained brain sections showing infarct size (white area). (C) Total infarction volume on d2 after tMCAO; *p = 0.0396, Student's *t-*test. (D) Average maximal infarction area from the slice with the largest infarction area per animal; *p = 0.0320, Student's *t-*test. (E) Biased body swing activity in 20 trials; **p = 0.0016, Mann-Whitney *U*-test. (F) Behavioral performance assessed by modified Bederson's score; *p = 0.0396, Mann-Whitney *U*-test.

Rats were acclimated to a complete diet made of refined ingredients for 6d. They were then divided into two groups, balanced for body weight and food intake during the acclimation period (Fig 3A). One group remained on the complete diet for 6d prior to stroke with average *ad libitum* daily food intake of 18.9 g/d. The second group was fed a protein-free diet for 6 d prior to stroke at the reduced amount of 11.4 g/d (∼40% calorie restriction). Although the initial aim was to normalize food intake among animals in the short-term protein-free DR group in expectation of food aversion to an incomplete (protein-free) diet, 36% of rats left some protein-free food uneaten, indicative of slightly greater aversion than predicted.

After preconditioning, a mild focal cortical stroke was induced by transient direct occlusion of the right MCA and bilateral CCAs with a suture for 60 min. One hour after reperfusion, body temperatures were not significantly elevated, consistent with milder lesion induction [41] (Fig S1). Two days after stroke induction, animals were sacrificed and infarction volumes were measured by TTC staining of brain sections. Rats in the protein-free DR group showed a 39% reduced infarction volume relative to the *ad libitum* fed group on the complete diet (Fig 3B, C). Similarly, the maximal infarction area was significantly reduced in the protein-free DR group compared to the complete diet group (Fig 3D). Prior to sacrifice 2d after stroke induction, rats in the protein-free diet group had smaller behavioral deficits as shown by biased body swing activity (Fig 3E) and Bederson's score (Fig 3F). Although plasma levels of total protein, albumin and urea were reduced in the protein-free DR group at the time of sacrifice consistent with the lack of protein in their diet, a number of other blood parameters including glucose, triglycerides, creatinine and prothrombin were not significantly different between diet groups (Table S2).

### Improved functional recovery from mild focal cortical stroke upon short-term protein-free DR

**Figure 4:**
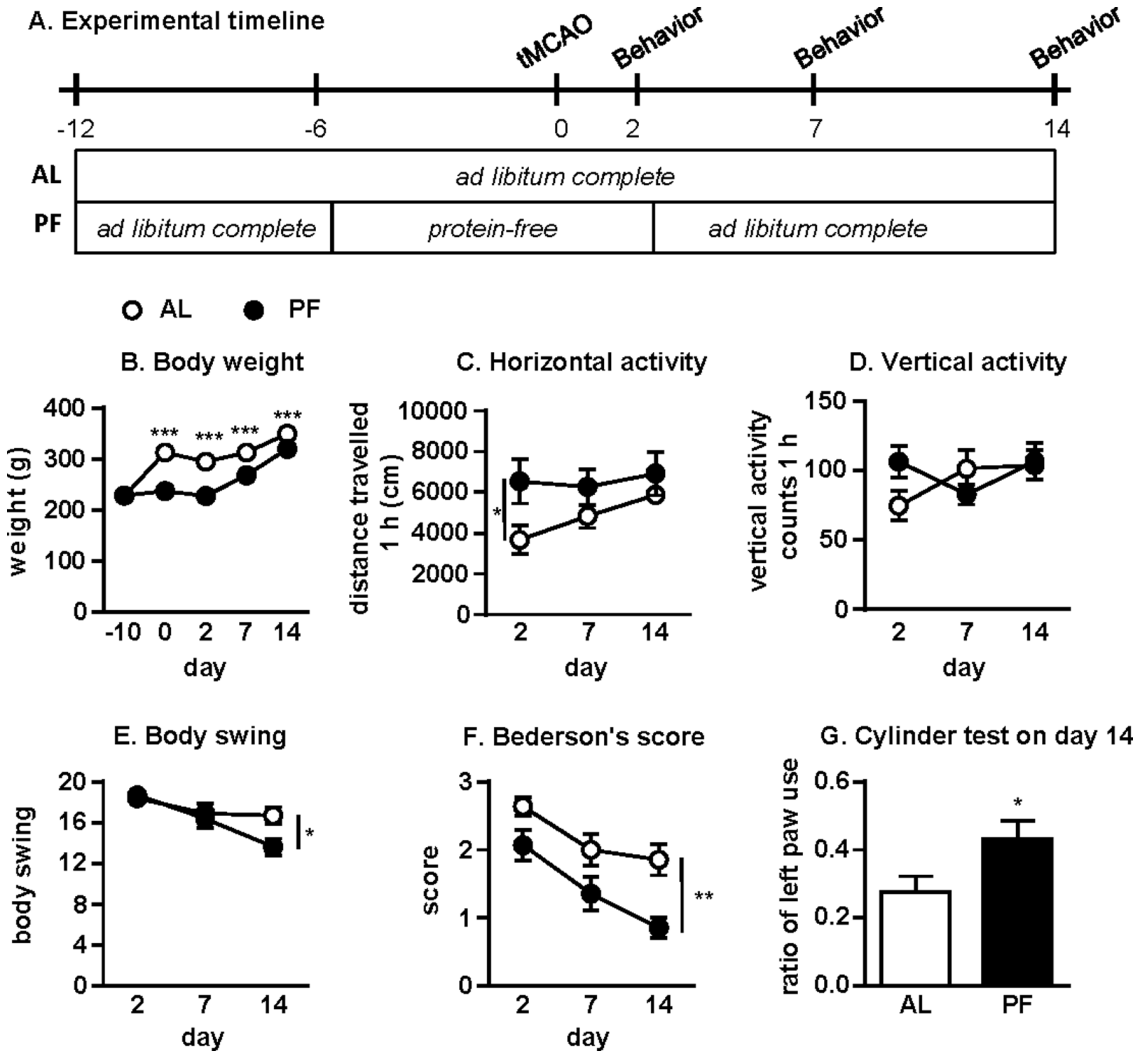
Protein-free DR promotes functional recovery after stroke. (A) Experimental timeline indicating periods of *ad libitum* access to a complete diet (AL, n = 14) or restricted access to a protein-free diet (PF, n = 14) relative to the onset of tMCAO on day 0 and subsequent behavioral testing on days 2, 7 and 14. (B) Average body weights on the indicated days relative to tMCAO on day 0; F_1,26_ = 96.20, ***p<0.0001, 2-way ANOVA. (C-G) Behavioral tests on the indicated days after tMCAO: (C) horizontal activity; F_1,26_ = 4.994, *p = 0.034, 2-way ANOVA; (D) vertical activity; F_1,26_ = 4.150, p = 0.052, 2-way ANOVA; (E) biased body swing activity in 20 trials; *p = 0.0211, Mann-Whitney *U*-test; (F) modified Bederson's score; **p = 0.0018, Mann-Whitney *U*-test; (G) cylinder test measured on d14 after tMCAO; *p = 0.0492, Mann-Whitney *U*-test.

We next assessed whether short-term protein-free DR could promote functional recovery up to 2 weeks after stroke. The experimental diets were applied as described above (Fig 4A). Short-term protein-free DR significantly reduced body weight (Fig 4B). Horizontal locomotor activity on d2 after stroke was significantly improved in the protein-free DR group, indicating faster recovery (Fig 4C). A similar tendency was observed with vertical activity on d2 but did not reach statistical significance (Fig 4D). Body swing activity was significantly reduced in the protein-free DR group on d14 after the stroke compared to the complete diet group (Fig 4E). Similarly, Bederson's score was significantly reduced on d14 (Fig 4F), with a similar trend on d2 and d7 (p = 0.0586 and p = 0.102, respectively, Mann-Whitney *U*-test). Rats in the protein-free DR group also showed improved performance in the cylinder test on d14 compared to the complete diet group (Fig 4G).

Finally, in order to shed light on underlying mechanism in comparison to the fasting paradigm, we performed qPCR analysis of gene expression changes in both cortex and striatum 24 hours post-stroke. As expected using this stroke model, gene expression changes in cortex were greater than in striatum. Nonetheless the patterns were similar between these two brain regions, including increased expression of neurotrophic factors BDNF and GDNF, stress response genes HMOX1 and GRP78, and inflammatory markers including ICAM1 and TNFRSF1A (Figure S2). However, with the exception of BDNF in the striatum, changes in expression of these genes in response to stroke were similar between diet groups, suggesting that protection afforded by fasting and protein-free DR could work via different mechanisms or time scale.

## Discussion

In hypoxia and global brain ischemia models, short-term water-only fasting is protective [34]–[38]. However, global brain hypoperfusion accounts for less than 10% of perioperative strokes in humans [2]. The majority (62%) of perioperative strokes are caused by focal ischemic insults that are mechanistically and pathologically different from hypoperfusion. Here we report that three days of preoperative water-only fasting reduced infarct volume compared to *ad libitum* fed rats in a focal stroke model involving forebrain and midbrain. In contrast to long-term DR and ischemic preconditioning, fasting did not increase baseline mRNA expression levels of cellular stress resistance genes such as the molecular chaperones HSP70 and GRP78 [39], or HMOX1 [42]. Nor did it significantly affect the expression of growth and neurotrophic factors including BDNF and FGF2 [39] or their downstream targets in the brain (Fig 2B, Table S1). Rather, increases in neurotrophic and growth factors and their receptors 24 hours after reperfusion in *ad libitum* fed animals correlated with increased infarct size. This is consistent with the notion that neurotrophic and growth factor upregulation is a relatively late event in ischemic brain damage, occurring downstream of innate immune system activation [43]. Our results are also in line with studies showing that intracranial applications of NTFs are in large part neuroprotective only when injected before the stroke, but are neither neuroprotective nor able to facilitate recovery when applied after the stroke [44]. Taken together, our results suggest that the mechanism of protection by long-term DR and short-term fasting in brain may differ, as has been suggested in other organs including the kidney [27].

In the severe stroke model involving forebrain and midbrain, protection afforded by fasting correlated with an altered inflammatory response. Ischemic injury to thalamic areas causes fever [45], which along with activation of microglia facilitates the expression of pro-inflammatory cytokines such as TNFα [46], [47]. Binding of TNFα to its receptor TNFRSF1A on endothelial cells induces ICAM1 expression [48], increasing blood-brain barrier permeability to infiltrating leukocytes and exacerbating tissue damage [49]. In line with the above, we observed a dramatic upregulation of TNFα in the infarcted right hemisphere of *ad libitum* fed animals 24 hours after reperfusion, whereas the expression of TNFα in the lesioned right hemisphere of fasted rats was not significantly affected. TNFRSF1A and ICAM1 expression in the striatum were also both significantly increased after tMCAO in *ad libitum* fed but not fasted rats. Since rats overexpressing TNFα are more susceptible to ischemic injury and mitochondrial dysfunction upon tMCAO [50], while TNFα neutralization is protective [51], our results suggest that suppression of TNFα expression upon focal ischemia reperfusion injury may be an important component of fasting-induced protection from focal stroke.

We also observed a significant increase in the levels of neutrophil chemoattractant CXCL1 in the plasma of rats following a 3d fast. Increased CXCL1 in the blood could reduce local inflammation by reducing the steepness of the chemokine gradient driving neutrophil chemotaxis from the vasculature to the site of brain injury, as well as by affecting adhesion molecule expression on neutrophils themselves [52]. Interestingly, in humans, short-term dietary preconditioning elevates serum levels of IL-8, the human paralogue of CXCL1 [53], warranting further study.

Several physiological parameters can also affect the outcome of experimental stroke. These include body temperature, blood glucose, blood pressure and blood gas levels. Hypothermia has been shown to effectively reduce ischemic injury in experimental models of brain injury [54], and body temperature was slightly but significantly reduced in fasted animals. However, therapeutic hypothermia in rodents and humans is protective only if body temperature is reduced by several degrees [55]. Because fasting reduced body temperature only by a half of a degree, fasting-associated neuroprotection is not likely to be a result of hypothermia. Hyperglycemia and hypoglycemia can both be deleterious after stroke [56], [57]. Although baseline blood glucose levels in fasted rats were reduced by 30% relative to the *ad libitum* fed group, this correlated with protection rather than susceptibility to ischemic brain damage. Finally, fasting in rats has been reported not to alter blood pressure or blood gas levels [37], thus making the above parameters unlikely to contribute significantly to protection.

Although water-only fasting is simple and effective in preclinical models of stroke, it may not be well-tolerated in some clinical settings. Thus, the nutritional basis of protection – whether an overall reduction in calories or the removal of specific nutrients – is important not only for understanding the underlying mechanism but also for evaluating the translational potential of the intervention. In fruit flies, the benefits of long-term DR on longevity can be abrogated by the addition of essential amino acids [58]. Long-term protein or individual amino acid restriction can also slow aging in rodents and precondition against acute stressors, including acetaminophen (paracetamol) toxicity [59] and ischemic injury to kidney and liver [28]. In studies of amino acid deficiency-mediated protection against renal and hepatic ischemia reperfusion injury, activation of the GCN2-dependent amino acid starvation response is required for protection in part through modulation of the systemic inflammatory response to injury and/or by activation of organ-autonomous stress resistance pathways [28]. Taken together, these data suggest broad evolutionary conservation of beneficial adaptive responses to protein/amino acid restriction.

Consistent with these reported benefits of protein/essential amino acid restriction, we found protection with a short-term protein-free DR regimen in a rat model of mild focal stroke, including reduced infarction volume and improved functional recovery. Interestingly, however, preliminary analyses of gene expression changes 24hr after reperfusion to probe candidate mechanisms of protection did not reveal overlap between the protein-free DR and fasting paradigms of neuroprotection. This could be due to different kinetics of gene expression in the severe vs. mild stroke models, or could indicate true differences in underlying mechanisms of protection by fasting and protein-free DR regimens. We focused our gene expression analysis on the 24hr time point in an attempt to uncover primary mechanisms of neuroprotection, as later time points could be confounded by differences in initial lesion size. Nonetheless, inflammatory responses after ischemic brain injury are also time dependent, beginning immediately after injury and lasting for months [60]. Thus, studies using unbiased gene expression analysis at multiple time-points and in different brain areas using gene arrays in combination with physiological, histological and behavioral parameters may help to dissect mechanisms involved in diet-induced neuroprotection and improved recovery after stroke in the future. How short-term dietary interventions such as fasting or short-term protein-free DR affect ischemic stress resistance in humans is currently unknown. However, there is substantial empirical and observational evidence that medically supervised fasting with periods of 7–21 days is efficacious in the treatment of rheumatic diseases, chronic pain syndromes, hypertension, and metabolic syndrome [61], [62]. Because metabolic responses to dietary restriction observed in experimental organisms are shared by humans [63], an expectation that benefits will translate to focal brain ischemia in humans is warranted [26]. The human functional equivalents of the 3d fasting or 6d protein-free DR regimens in rodents tested here are not known, but represent an important next question in translation to the clinic. In conclusion, due to its simplicity, cost-effectiveness and presumed low risk, short-term dietary preconditioning may carry an immediate potential for clinical application in perioperative risk management.

## Supporting Information

Figure S1 **Rectal body temperature in rat cortical stroke model in** ***ad libitum*** **complete (AL, n = 4) and protein free (PF, n = 4) diet groups before and 1 h after reperfusion.**
(TIF)

Figure S2 **Expression of selected genes 24 hours after tMCAO in the unlesioned left (L) and lesioned right (R) cortices and striata of rats in** ***ad libitum*** **(AL, n = 8) and protein-free (PF, n = 7) diet groups, measured by qPCR and expressed relative to the unlesioned AL group.** Asterisks indicate difference between the lesioned and unlesioned hemispheres in the same diet group. No significant differences were observed between diet groups; *p<0.05; **p<0.01; ***p<0.001, 1-way ANOVA.
(TIF)

Methods S1 **Supplementary Methods.**
(DOCX)

Table S1 (PDF)

Table S2 **Analysis of the indicated component from blood plasma from rats fed a complete diet** ***ad libitum*** **(AL) or a protein free (PF) diet for 6 days prior to and 2 days after induction of cortical stroke.**
(DOCX)

## References

[1] Kellermann K, Jungwirth B (2010) Avoiding stroke during cardiac surgery. Semin Cardiothorac Vasc Anesth 14: 95–101. 20478949 10.1177/1089253210370902

[2] Selim M (2007) Perioperative stroke. N Engl J Med 356: 706–713. 17301301 10.1056/NEJMra062668

[3] Hall MJ, DeFrances CJ, Williams SN, Golosinskiy A, Schwartzman A (2010) National Hospital Discharge Survey: 2007 summary. Natl Health Stat Report: 1–20, 24. 21086860

[4] Poldermans D, Bax JJ, Boersma E, De Hert S, Eeckhout E, et al (2009) Guidelines for pre-operative cardiac risk assessment and perioperative cardiac management in non-cardiac surgery. Eur Heart J 30: 2769–2812. 19713421 10.1093/eurheartj/ehp337

[5] Newman MF, Mathew JP, Grocott HP, Mackensen GB, Monk T, et al (2006) Central nervous system injury associated with cardiac surgery. Lancet 368: 694–703. 16920475 10.1016/S0140-6736(06)69254-4

[6] Bruggemans EF (2013) Cognitive dysfunction after cardiac surgery: Pathophysiological mechanisms and preventive strategies. Neth Heart J 21: 70–73. 23184600 10.1007/s12471-012-0347-x PMC3547425

[7] Green AR (2008) Pharmacological approaches to acute ischaemic stroke: reperfusion certainly, neuroprotection possibly. Br J Pharmacol 153 Suppl 1 S325–338. 18059324 10.1038/sj.bjp.0707594 PMC2268079

[8] Murry CE, Jennings RB, Reimer KA (1986) Preconditioning with ischemia: a delay of lethal cell injury in ischemic myocardium. Circulation 74: 1124–1136. 3769170 10.1161/01.cir.74.5.1124

[9] Dirnagl U, Becker K, Meisel A (2009) Preconditioning and tolerance against cerebral ischaemia: from experimental strategies to clinical use. Lancet neurology 8: 398–412. 19296922 10.1016/S1474-4422(09)70054-7 PMC2668955

[10] Bowen KK, Naylor M, Vemuganti R (2006) Prevention of inflammation is a mechanism of preconditioning-induced neuroprotection against focal cerebral ischemia. Neurochemistry international 49: 127–135. 16759752 10.1016/j.neuint.2006.02.011

[11] Sharp FR, Ran R, Lu A, Tang Y, Strauss KI, et al (2004) Hypoxic preconditioning protects against ischemic brain injury. NeuroRx 1: 26–35. 15717005 10.1602/neurorx.1.1.26 PMC534910

[12] Rosenzweig HL, Minami M, Lessov NS, Coste SC, Stevens SL, et al (2007) Endotoxin preconditioning protects against the cytotoxic effects of TNFalpha after stroke: a novel role for TNFalpha in LPS-ischemic tolerance. J Cereb Blood Flow Metab 27: 1663–1674. 17327883 10.1038/sj.jcbfm.9600464

[13] Perdrizet GA, Heffron TG, Buckingham FC, Salciunas PJ, Gaber AO, et al (1989) Stress conditioning: a novel approach to organ preservation. Curr Surg 46: 23–26. 2656107

[14] Speakman JR, Mitchell SE (2011) Caloric restriction. Mol Aspects Med 32: 159–221. 21840335 10.1016/j.mam.2011.07.001

[15] Yu ZF, Mattson MP (1999) Dietary restriction and 2-deoxyglucose administration reduce focal ischemic brain damage and improve behavioral outcome: evidence for a preconditioning mechanism. J Neurosci Res 57: 830–839. 10467254

[16] Duan W, Guo Z, Mattson MP (2001) Brain-derived neurotrophic factor mediates an excitoprotective effect of dietary restriction in mice. J Neurochem 76: 619–626. 11208925 10.1046/j.1471-4159.2001.00071.x

[17] Duan W, Lee J, Guo Z, Mattson MP (2001) Dietary restriction stimulates BDNF production in the brain and thereby protects neurons against excitotoxic injury. Journal of molecular neuroscience: MN 16: 1–12. 11345515 10.1385/JMN:16:1:1

[18] Anson RM, Guo Z, de Cabo R, Iyun T, Rios M, et al (2003) Intermittent fasting dissociates beneficial effects of dietary restriction on glucose metabolism and neuronal resistance to injury from calorie intake. Proc Natl Acad Sci U S A 100: 6216–6220. 12724520 10.1073/pnas.1035720100 PMC156352

[19] Lee J, Duan W, Long JM, Ingram DK, Mattson MP (2000) Dietary restriction increases the number of newly generated neural cells, and induces BDNF expression, in the dentate gyrus of rats. Journal of molecular neuroscience: MN 15: 99–108. 11220789 10.1385/JMN:15:2:99

[20] Lee J, Duan W, Mattson MP (2002) Evidence that brain-derived neurotrophic factor is required for basal neurogenesis and mediates, in part, the enhancement of neurogenesis by dietary restriction in the hippocampus of adult mice. Journal of neurochemistry 82: 1367–1375. 12354284 10.1046/j.1471-4159.2002.01085.x

[21] Ferrer I, Krupinski J, Goutan E, Marti E, Ambrosio S, et al (2001) Brain-derived neurotrophic factor reduces cortical cell death by ischemia after middle cerebral artery occlusion in the rat. Acta Neuropathol 101: 229–238. 11307622 10.1007/s004010000268

[22] Schabitz WR, Schwab S, Spranger M, Hacke W (1997) Intraventricular brain-derived neurotrophic factor reduces infarct size after focal cerebral ischemia in rats. J Cereb Blood Flow Metab 17: 500–506. 9183287 10.1097/00004647-199705000-00003

[23] Chandrasekar B, Nelson JF, Colston JT, Freeman GL (2001) Calorie restriction attenuates inflammatory responses to myocardial ischemia-reperfusion injury. Am J Physiol Heart Circ Physiol 280: H2094–2102. 11299211 10.1152/ajpheart.2001.280.5.H2094

[24] Ahmet I, Wan R, Mattson MP, Lakatta EG, Talan M (2005) Cardioprotection by intermittent fasting in rats. Circulation 112: 3115–3121. 16275865 10.1161/CIRCULATIONAHA.105.563817

[25] Mitchell JR, Beckman JA, Nguyen LL, Ozaki CK (2013) Reducing elective vascular surgery perioperative risk with brief preoperative dietary restriction. Surgery 153: 594–598. 23218877 10.1016/j.surg.2012.09.007 PMC3602128

[26] Robertson LT, Mitchell JR (2013) Benefits of short-term dietary restriction in mammals. Exp Gerontol 48: 1043–1048. 23376627 10.1016/j.exger.2013.01.009 PMC3745522

[27] Mitchell JR, Verweij M, Brand K, van de Ven M, Goemaere N, et al (2010) Short-term dietary restriction and fasting precondition against ischemia reperfusion injury in mice. Aging Cell 9: 40–53. 19878145 10.1111/j.1474-9726.2009.00532.x PMC3412229

[28] Peng W, Robertson L, Gallinetti J, Mejia P, Vose S, et al (2012) Surgical stress resistance induced by single amino acid deprivation requires Gcn2 in mice. Sci Transl Med 4: 118ra111. 10.1126/scitranslmed.3002629 PMC3535286 22277968

[29] Koizumi J, Yoshida Y, Nakazawa T, Ooneda G (1986) Experimental studies of ischemic brain edema I: A new experimental model of cerebral embolism in rats in which recirculation can be introduced in the ischemic area. Jpn J Stroke 8: 1–8.

[30] Lee ST, Chu K, Jung KH, Kim JH, Huh JY, et al (2012) miR-206 regulates brain-derived neurotrophic factor in Alzheimer disease model. Ann Neurol 72: 269–277. 22926857 10.1002/ana.23588

[31] Airavaara M, Shen H, Kuo CC, Peranen J, Saarma M, et al (2009) Mesencephalic astrocyte-derived neurotrophic factor reduces ischemic brain injury and promotes behavioral recovery in rats. J Comp Neurol 515: 116–124. 19399876 10.1002/cne.22039 PMC2723810

[32] Miura P, Amirouche A, Clow C, Belanger G, Jasmin BJ (2012) Brain-derived neurotrophic factor expression is repressed during myogenic differentiation by miR-206. J Neurochem 120: 230–238. 22081998 10.1111/j.1471-4159.2011.07583.x

[33] Bederson JB, Pitts LH, Tsuji M, Nishimura MC, Davis RL, et al (1986) Rat middle cerebral artery occlusion: evaluation of the model and development of a neurologic examination. Stroke 17: 472–476. 3715945 10.1161/01.str.17.3.472

[34] Kirsch JR, D'Alecy LG (1979) Effect of altered availability of energy-yielding substrates upon survival from hypoxia in mice. Stroke 10: 288–291. 462515 10.1161/01.str.10.3.288

[35] Combs DJ, D'Alecy LG (1987) Motor performance in rats exposed to severe forebrain ischemia: effect of fasting and 1,3-butanediol. Stroke 18: 503–511. 3564110 10.1161/01.str.18.2.503

[36] Rehncrona S, Rosen I, Smith ML (1985) Effect of different degrees of brain ischemia and tissue lactic acidosis on the short-term recovery of neurophysiologic and metabolic variables. Exp Neurol 87: 458–473. 3972049 10.1016/0014-4886(85)90176-1

[37] Marie C, Bralet AM, Gueldry S, Bralet J (1990) Fasting prior to transient cerebral ischemia reduces delayed neuronal necrosis. Metab Brain Dis 5: 65–75. 2385215 10.1007/BF01001047

[38] Go KG, Prenen GH, Korf J (1988) Protective effect of fasting upon cerebral hypoxic-ischemic injury. Metab Brain Dis 3: 257–263. 3241603 10.1007/BF00999535

[39] Arumugam TV, Phillips TM, Cheng A, Morrell CH, Mattson MP, et al (2010) Age and energy intake interact to modify cell stress pathways and stroke outcome. Annals of neurology 67: 41–52. 20186857 10.1002/ana.21798 PMC2844782

[40] Chen ST, Hsu CY, Hogan EL, Maricq H, Balentine JD (1986) A model of focal ischemic stroke in the rat: reproducible extensive cortical infarction. Stroke 17: 738–743. 2943059 10.1161/01.str.17.4.738

[41] Lin TN, Chen ST, He YY, Cheung WM, Hsu CY (2009) Three-Vessel Middle Cerebral Artery Occlusion Model. In: Chen J, editor. Animal Models of Acute Neurological Injuries. New York: Humana Press. pp. 141–153.

[42] Zeynalov E, Shah ZA, Li RC, Dore S (2009) Heme oxygenase 1 is associated with ischemic preconditioning-induced protection against brain ischemia. Neurobiol Dis 35: 264–269. 19465127 10.1016/j.nbd.2009.05.010 PMC2740718

[43] Yenari MA, Kauppinen TM, Swanson RA (2010) Microglial activation in stroke: therapeutic targets. Neurotherapeutics 7: 378–391. 20880502 10.1016/j.nurt.2010.07.005 PMC5084300

[44] Harvey BK, Hoffer BJ, Wang Y (2005) Stroke and TGF-beta proteins: glial cell line-derived neurotrophic factor and bone morphogenetic protein. Pharmacol Ther 105: 113–125. 15670622 10.1016/j.pharmthera.2004.09.003

[45] Reglodi D, Somogyvari-Vigh A, Maderdrut JL, Vigh S, Arimura A (2000) Postischemic spontaneous hyperthermia and its effects in middle cerebral artery occlusion in the rat. Exp Neurol 163: 399–407. 10833314 10.1006/exnr.2000.7367

[46] Damm J, Wiegand F, Harden LM, Gerstberger R, Rummel C, et al (2012) Fever, sickness behavior, and expression of inflammatory genes in the hypothalamus after systemic and localized subcutaneous stimulation of rats with the Toll-like receptor 7 agonist imiquimod. Neuroscience 201: 166–183. 22116053 10.1016/j.neuroscience.2011.11.013

[47] Gregersen R, Lambertsen K, Finsen B (2000) Microglia and macrophages are the major source of tumor necrosis factor in permanent middle cerebral artery occlusion in mice. J Cereb Blood Flow Metab 20: 53–65. 10616793 10.1097/00004647-200001000-00009

[48] Yang L, Froio RM, Sciuto TE, Dvorak AM, Alon R, et al (2005) ICAM-1 regulates neutrophil adhesion and transcellular migration of TNF-alpha-activated vascular endothelium under flow. Blood 106: 584–592. 15811956 10.1182/blood-2004-12-4942 PMC1635241

[49] Amantea D, Nappi G, Bernardi G, Bagetta G, Corasaniti MT (2009) Post-ischemic brain damage: pathophysiology and role of inflammatory mediators. FEBS J 276: 13–26. 19087196 10.1111/j.1742-4658.2008.06766.x

[50] Pandya JD, Sullivan PG, Pettigrew LC (2011) Focal cerebral ischemia and mitochondrial dysfunction in the TNFalpha-transgenic rat. Brain Res 1384: 151–160. 21300036 10.1016/j.brainres.2011.01.102 PMC3471782

[51] Barone FC, Arvin B, White RF, Miller A, Webb CL, et al (1997) Tumor necrosis factor-alpha. A mediator of focal ischemic brain injury. Stroke 28: 1233–1244. 9183357 10.1161/01.str.28.6.1233

[52] Simonet WS, Hughes TM, Nguyen HQ, Trebasky LD, Danilenko DM, et al (1994) Long-term impaired neutrophil migration in mice overexpressing human interleukin-8. J Clin Invest 94: 1310–1319. 7521886 10.1172/JCI117450 PMC295217

[53] van Ginhoven TM, Dik WA, Mitchell JR, Smits-Te Nijenhuis MA, van Holten-Neelen C, et al.. (2010) Dietary Restriction Modifies Certain Aspects of the Postoperative Acute Phase Response. J Surg Res. 10.1016/j.jss.2010.03.038 20538300

[54] Varon J, Acosta P (2008) Therapeutic hypothermia: past, present, and future. Chest 133: 1267–1274. 18460529 10.1378/chest.07-2190

[55] Kollmar R, Blank T, Han JL, Georgiadis D, Schwab S (2007) Different degrees of hypothermia after experimental stroke: short- and long-term outcome. Stroke 38: 1585–1589. 17363720 10.1161/STROKEAHA.106.475897

[56] Gandhi GY, Nuttall GA, Abel MD, Mullany CJ, Schaff HV, et al (2005) Intraoperative hyperglycemia and perioperative outcomes in cardiac surgery patients. Mayo Clin Proc 80: 862–866. 16007890 10.4065/80.7.862

[57] Kagansky N, Levy S, Knobler H (2001) The role of hyperglycemia in acute stroke. Arch Neurol 58: 1209–1212. 11493160 10.1001/archneur.58.8.1209

[58] Grandison RC, Piper MD, Partridge L (2009) Amino-acid imbalance explains extension of lifespan by dietary restriction in Drosophila. Nature 462: 1061–1064. 19956092 10.1038/nature08619 PMC2798000

[59] Miller RA, Buehner G, Chang Y, Harper JM, Sigler R, et al (2005) Methionine-deficient diet extends mouse lifespan, slows immune and lens aging, alters glucose, T4, IGF-I and insulin levels, and increases hepatocyte MIF levels and stress resistance. Aging Cell 4: 119–125. 15924568 10.1111/j.1474-9726.2005.00152.x PMC7159399

[60] Thored P, Heldmann U, Gomes-Leal W, Gisler R, Darsalia V, et al (2009) Long-term accumulation of microglia with proneurogenic phenotype concomitant with persistent neurogenesis in adult subventricular zone after stroke. Glia 57: 835–849. 19053043 10.1002/glia.20810

[61] Michalsen A, Li C (2013) Fasting therapy for treating and preventing disease - current state of evidence. Forsch Komplementmed 20: 444–453. 24434759 10.1159/000357765

[62] Fond G, Macgregor A, Leboyer M, Michalsen A (2013) Fasting in mood disorders: neurobiology and effectiveness. A review of the literature. Psychiatry Res 209: 253–258. 23332541 10.1016/j.psychres.2012.12.018

[63] Heilbronn LK, de Jonge L, Frisard MI, DeLany JP, Larson-Meyer DE, et al (2006) Effect of 6-month calorie restriction on biomarkers of longevity, metabolic adaptation, and oxidative stress in overweight individuals: a randomized controlled trial. Jama 295: 1539–1548. 16595757 10.1001/jama.295.13.1539 PMC2692623

