# Supplementary figures and images for: Short-term Preoperative Dietary Restriction Is Neuroprotective in a Rat Focal Stroke Model
Source: PLoS One. 2014 Apr 4;9(4):e93911. doi: 10.1371/journal.pone.0093911 (PMC3976327; doi:10.1371/journal.pone.0093911)

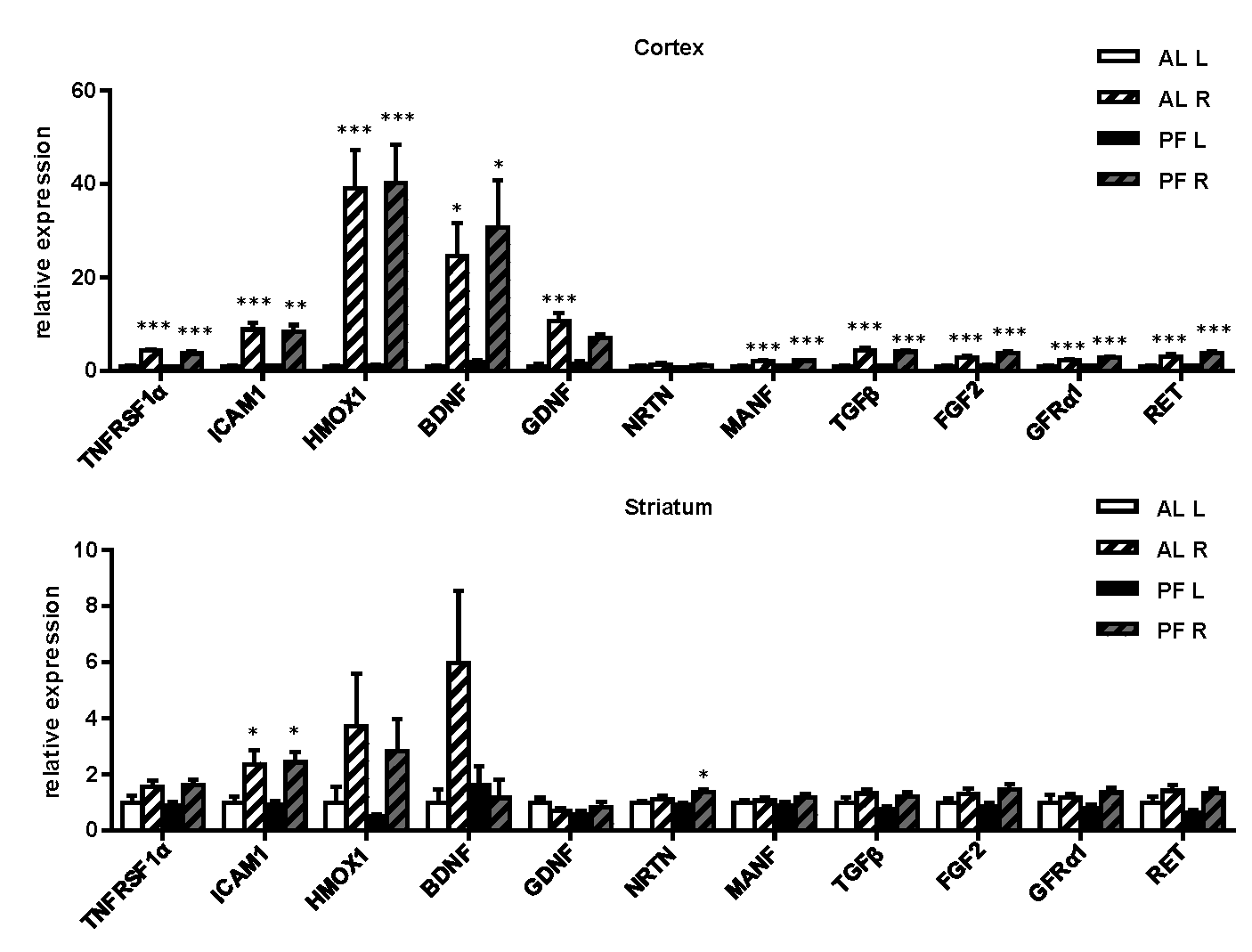

Supplement: Figure S2 — Expression of selected genes 24 hours after tMCAO in the unlesioned left (L) and lesioned right (R) cortices and striata of rats in ad libitum (AL, n = 8) and protein-free (PF, n = 7) diet groups, measured by qPCR and expressed relative to the unlesioned AL group. Asterisks indicate difference between the lesioned and unlesioned hemispheres in the same diet group. No significant differences were observed between diet groups; *p<0.05; **p<0.01; ***p<0.001, 1-way ANOVA. (TIF) [file pone.0093911.s002.tif]
