# Supplementary material for: Short-term Preoperative Dietary Restriction Is Neuroprotective in a Rat Focal Stroke Model
Source: PLoS One. 2014 Apr 4;9(4):e93911. doi: 10.1371/journal.pone.0093911 (PMC3976327; doi:10.1371/journal.pone.0093911)
Supplement: Methods S1 — Supplementary Methods. (DOCX) [file pone.0093911.s003.docx]

**Supplementary Methods**

**Animals**

All animal experiments were carried out according to the National Institute of Health (NIH) guidelines for the care and use of laboratory animals. Fasting in combination with intraluminal filament-induced stroke experiments were conducted at Charles River Laboratories (CRL) LTD, Kuopio, Finland and approved by the Ethical Committee of the National Laboratory Animal Center, Kuopio, Finland. Protein-free diet in combination with suture-induced stroke experiments were conducted at the University of Helsinki (UH), Institute of Biotechnology and Laboratory Animal Centre and approved by the National Animal Experiment Board (ELLA). Adult male Sprague-Dawley (SD) rats weighing 240-300 g used in all the experiments were housed at standard temperature (22 ± 1°C) in a light-controlled environment (lights on from 7 AM to 7 PM). Rats had *ad libitum* access to food and water unless indicated otherwise.

**Diets**

Diets used in the fasting experiments (Teklad Global 2016, Harlan Laboratories) were wheat/corn/soy based and consisted of 22% calories from protein, 66% from carbohydrate and 12% from fat. Diets made of refined ingredients used in the protein-free DR experiments were purchased from Research Diets (New Brunswick, NJ, USA). The complete diet (D12450B) consisted of 18% calories from protein, 72% from carbohydrate and 10% from fat. 1055.05g of the diet consisted of 200g casein, 3g L-cystine, 315g corn starch, 35g maltodextrin 10, 350g sucrose, 50g cellulose, 25g soybean oil, 20g lard, 10g mineral mix S10026, 13g dicalcium phosphate, 5.5g calcium carbonate, 16.5g potassium citrate, 10g vitamin mix V10001, 2g choline bitartrate and 0.05g FD&C yellow dye #5. The protein free diet (D08043003) consisted of 90% calories from carbohydrate and 10% from fat. 1031.05g of the diet consisted of 344g corn starch, 35g maltodextrin 10, 500g sucrose, 50g cellulose BW200, 25g soybean oil, 20g lard, 10g mineral mix S10026, 13g dicalcium phosphate, 5.5g calcium carbonate, 16.5g potassium citrate, 10g vitamin mix V10001, 2g choline bitartrate and 0.05g FD&C red dye #40.

**Surgical procedures**

Two different surgical procedures were used in the study. A severe focal stroke involving forebrain and midbrain was induced by occlusion of the middle cerebral artery (MCA) for 60 min with an intraluminal filament. A milder focal stroke involving cortex only was induced by transient direct occlusion of the right MCA and bilateral CCAs with a 10-0 suture for 60 min followed by reperfusion as described in greater detail below:

*Intraluminal filament-induced stroke:* Severe stroke induction in conjunction with fasting was performed at Charles River Laboratories LTD (CRL), Kuopio, Finland, a professional provider of the intraluminal suture occlusion model to academic and industrial clients. Focal cerebral ischemia resulting in lesion in the forebrain and midbrain was produced by transient MCA occlusion (tMCAO) in male SD rats according to Koizumi with modifications[[1](#_ENREF_1)]. Briefly, rats were anesthetized with isoflurane (Halocarbon Laboratories) in a 70%/30% N_2_O/O_2_ mix flowing at 300 mL/min. Body temperature was maintained at 37°C during all surgical procedures. After midline skin incision, the right common carotid artery (CCA) was exposed, and the external carotid artery (ECA) was ligated distal from the carotid bifurcation. A 0.25-mm diameter monofilament nylon thread (Genzyme), with tip blunted, was inserted 22-23 mm into the internal carotid artery (ICA) up to the origin of the MCA. The wound was temporarily closed and rats were allowed to recover from anesthesia. After 60 min of ischemia, rats were re-anesthetized and reperfused by removal of the filament from the MCA, followed by wound closing and disinfection. After recovery from anesthesia in homeothermic cages, rats were returned to their home cages. To determine whether the MCA occlusion surgery produced a lesion, core body temperature and deficits in behavior were measured. Stroke involving thalamic areas is associated with fever[[2](#_ENREF_2)] and the presence of fever was confirmed in each animal one hour after reperfusion. Within each experiment, the control group and the treated group were from the same batch of animals and treated in a “blind” fashion.

*10-0 suture-induced cortical stroke:* Mild stroke induction in conjunction with protein-free DR was performed at the University of Helsinki, Helsinki, Finland. Focal cerebral ischemia resulting cortical-only stroke was produced by ligation of the right MCA and bilateral CCAs as described previously[[3-5](#_ENREF_3)]. Briefly, animals were anesthetized with chloral hydrate (0.4 g/kg, i.p.). The bilateral CCAs were identified and isolated through a ventral midline cervical incision. Rats were placed in a stereotaxic apparatus and a craniotomy was performed on the right hemisphere. The right MCA was ligated with a 10-0 suture and bilateral CCAs were ligated with non-traumatic arterial clamps for 60 minutes. Following ischemia, reperfusion was initiated by suture and clamp removal. After recovery from anesthesia in homeothermic cages, rats were returned to their home cages. To prevent dehydration, rats were injected with saline (4-5 ml per rat) and monitored for possible post-operative complications. Cortical-only stroke does not result in fever in rats[[6](#_ENREF_6)] (Supplementary Fig S1) thus the presence of lesion was verified by behavioral tests and TTC staining 48 hours after stroke.

**Behavioral procedures**

All tests were conducted by an investigator blinded to the treatment groups. Body asymmetry in 10-0 suture induced cortical stroke model was analyzed using an elevated body swing test and cylinder test. In the body swing test rats were examined for lateral movements/turning when their bodies were suspended 20 cm above the testing table by lifting from the base of the tail. The frequency of initial turning of the head or upper body contralateral to the ischemic side was counted in 20 consecutive trials. The maximum impairment in body asymmetry is 20 contralateral turns per 20 trials. Neurological deficits were evaluated using Bederson’s score[[7](#_ENREF_7)]. In a postural reflex test, rats were examined for the degree of abnormal posture when suspended 20-30 cm above the testing table and scored according to the following criteria: 0, Rats extend both forelimbs straight with no observable deficits; 1, Rats keep one forelimb to the breast and extend the other forelimb straight; 2, Rats show decreased resistance to lateral push in addition to behavior in score 1 without circling; 3, Rats twist the upper half of their body in addition to behavior in score 2. In the cylinder test, rats were placed in a plexiglass cylinder and the frequency of forepaw exploratory touches using left (impaired) vs. right forepaw in the first 20 touches was measured. Locomotor activity was measured using an infrared activity monitor (MedAssociates Inc.). Animals were individually placed in a 43x43x33 cm plexiglass open box containing horizontal infrared sensors. Locomotor activity was measured for a period of 1h.

**Analysis of infarction volume**

*Intraluminal filament-induced forebrain and midbrain stroke:* Seven days after tMCAO, rats were deeply anesthetized with pentobarbital (60 mg/kg Mebunat, Orion Pharma, Finland) and perfused transcardially with heparinized (2.5 IU/ml; Leo Pharma) saline followed by 4 % paraformaldehyde (PFA) in PBS, and post-fixed in PFA for 24 hours. Brains were rinsed with PBS and embedded in perfluoropolyether (FOMBLIN). T2 and T2* -weighted MRI was performed with the use of a Varian Inova console interfaced to a 4.7T horizontal magnet equipped with actively shielded gradient coils (Magnex Scientific Ltd, Abington, UK). A half-volume coil, driven in quadrature mode, was used for signal transmission and reception. For determination of infarct volume, T2-weighted multi-slice (12-14 continuous slices) images were acquired using double spin-echo sequence with adiabatic refocusing pulses TR = 3 s, TE = 80 ms, matrix size of 256x128, FOV of 35*35 mm^2^, and a slice thickness of 1 mm. Lesion quantitation was done by manually delineating lesion outlines from MR images based on T2 contrast between lesioned and healthy tissue obtained from coronal images at 1-mm intervals. Image analyses were performed using MATLAB software by an observer blinded to the treatment groups. Measurements were performed at CRL LTD, Kuopio, Finland, a professional provider of the intraluminal suture occlusion model to academic and industrial clients.

*10-0 suture-induced cortical stroke:* Triphenyltetrazolium chloride (TTC) staining was performed 2 days after tMCAO, as described previously[[5](#_ENREF_5)]. Rats were decapitated and the brains were removed and sliced into 2-mm-thick sections using an acrylic rat brain block. The brain slices were incubated in a 2 % TTC solution (Sigma, St. Louis, MO, USA) for 15 min at room temperature and then transferred into a 4 % paraformaldehyde solution for fixation. The area of infarction in each slice was measured with a digital scanner and ImageJ software (http://rsb.info.nih.gov/ij/index.html). The volume of infarction in each animal was quantified by an observer blinded to the treatment groups and obtained from the product of average slice thickness (2 mm) and sum of infarction areas in all brain slices examined.

**Blood and plasma measurements**

Glucose levels were measured from fresh venous blood with a Glucocard II Super device (Akray Factory Inc., Shiga, Japan) at CRL, Kuopio, Finland. Blood samples were collected into pre-cooled polyethylene tubes containing K_2_-EDTA as anticoagulant and kept on ice until centrifugation at 2000 g (4°C). Plasma was snap frozen in liquid nitrogen. For cytokine analysis, plasma from 0 and 4 hours after tMCAO was either used directly or diluted 1:2 and analyzed on the Rat Demonstration Multi-Spot plate (Meso Scale Discovery, Gaithersburg, MD) for the presence of chemokine (C-X-C motif) ligand 1 (CXCL1), interleukin-1 beta (IL-1β), interleukin-4 (IL-4), interleukin-5 (IL-5), tumor necrosis factor alpha (TNFα), interferon alpha (IFNα) and interleukin-13 (IL-13) according to the manufacturer’s instructions at Harvard School of Public Health, Boston, US. Clinical chemistry analyses of alanine transaminase (ALAT), protein, albumin, urea, creatinine, glucose, triglycerides, sodium and chloride were performed with an automatic analyzer according to manufacturer’s instructions (Konelab 30i, Thermo Fisher Scientific, Vantaa, Finland) and prothrombin was measured using a Rat Prothrombin ELISA kit (Shanghai BlueGene Biotech Co., LTD), according to the manufacturer’s instructions at the UH, Helsinki, Finland.

**Real-time quantitative PCR**

For real-time quantitative PCR (qPCR), rats were sacrificed under isoflurane anesthesia. Right and left hemispheres were immediately dissected on ice, snap frozen in liquid nitrogen and stored at -80°C at CRL LTD, Kuopio, Finland prior transportation to UH, Helsinki, Finland. Total RNA was extracted from cortex or striatum using TRIzol reagent (Life Technologies) and stored at -80°C until processed for reverse transcription. Total RNA concentration and quality were determined with NanoDrop 1000 v3.7.1 and Agilent 2100 Bioanalyzer. Samples were treated with DNase I (Ambion, Inc.) to prevent contamination with genomic DNA. cDNA for qPCR analysis was synthesized from 100 ng of total RNA using Transcriptor reverse transcriptase and random hexamer primers as recommended by the manufacturer (Roche Diagnostics). The cDNA product was stored at -20°C or used immediately for qPCR. Primers for the qPCR reactions were designed using Primer3 primer design algorithm[[8](#_ENREF_8)] and are shown in Supplementary Table S1. qPCR was performed with Lightcycler® 480 real-time PCR system (Roche Diagnostics) using Lightcycler® 480 SYBR Green I Master complemented with 2.5 pmol of primers and cDNA corresponding to 1.25 ng of total RNA in the final volume of 10 μl on 384-well plates. Three replicates of each reaction were included in the qPCR runs. The results were analyzed with Lightcycler® 480 Software Release 1.5.0 SP1. Gene expression was normalized to peptidylprolyl isomerase A. For each primer pair, primer efficiencies were determined and used for gene expression analysis. Calculation method based on primer efficiencies is available upon request. Real-time qPCR analysis was performed at UH, Helsinki, Finland.

**Neuroprotection against severe focal stroke by preoperative fasting (Figure 1)**

In hypoxia and global brain ischemia models, short-term water-only fasting is protective[[9-13](#_ENREF_9)]. The purpose of this experiment was to establish whether water-only fasting is neuroprotective in a focal stroke model in rats. The filament tMCAO model was selected as it produces a massive infarction localized both in the striatum and cortex. We used an experimental design with the between-subjects factor of food dose (*ad libitum*, water-only fasting) and the within-subjects factor of day. The control group (n=11) had continuous *ad libitum* access to facility chow. The pre-stroke fasting group (n=14) had *ad libitum* access to chow until 3 days before the stroke. All animals had *ad libitum* access to drinking water throughout the study period. After stroke, the water-only fasted rats regained *ad libitum* access to food.

**Suppression of pro-inflammatory response to severe focal stroke by fasting (Figure 2)**

In order to investigate the molecular mechanism of fasting-induced neuroprotection, we examined gene expression in the brain prior to and 24 hours after tMCAO. To discriminate the possible effects of surgery from those of stroke, sham-operated animals undergoing all surgical procedures except MCA occlusion were included as controls. To this end, rats were divided into 6 groups (n=5-6/group): Groups 1 and 2 had *ad libitum* access to food or were fasted for 3 days and sacrificed prior to surgery for baseline measurements; Groups 3 and 4 had *ad libitum* access to food or were fasted for 3 days prior to sham operation and sacrificed 24hr after surgery; Groups 5 and 6 had *ad libitum* access to food or were fasted for 3 days prior to tMCAO and sacrificed 24hr after tMCAO.

For each animal undergoing tMCAO, the contralateral (non-lesioned) hemisphere was used as an internal control. Striatal gene expression was analyzed using qPCR. Genes were selected based on their known or presumed involvement in focal stroke pathogenesis, neuroprotective effect and/or differential regulation upon DR according to the following categories: neurotrophic and growth factors/receptors, inflammation, cellular stress resistance and cell death. Genes included in the analysis are listed in Supplementary Table S1. To test the systemic effects of water-only fasting on immune system activation, blood plasma samples from *ad libitum* fed and fasted animals were collected at baseline and 4 hours after tMCAO for cytokine analysis.

**Neuroprotection against mild focal cortical stroke by protein-free dietary restriction (Figure 3)**

The purpose of this experiment was to determine whether a milder preconditioning regimen consisting of a protein-free diet promotes neuroprotection in a milder cortical-only stroke model. Our objective was also to assess infarct size at its maximum, i.e. 48h after stroke and evaluate functional outcome at 2 days post-stroke. We used an experimental design with the between-subjects factor of diet (complete diet, protein-free DR). Individually housed rats were acclimated on a complete diet (Research Diets, Inc, USA, D12450B, 3.85 kcal/g) for 6 days while food intake and body weight were measured. Rats were then divided into two groups: the first (n=15) was continued on the complete diet, and the second (n=14) switched to a protein-free diet (Research Diets, Inc., USA, D08043003, 3.85 kcal/g). Because nutritionally incomplete (e.g. protein-free) diets are known to provoke food aversion in rodents, a well-documented but incompletely understood phenomenon[[14-16](#_ENREF_14)], the protein-free diet was provided at 60% of the average daily intake of the complete diet over the baseline period in an attempt to normalize food intake within this group. Rats were maintained on the experimental diets for 6 days prior to and 2 days after tMCAO. Infarction volume measurements and behavioral tests were performed on day 2 after the stroke. Plasma was collected 2 days after tMCAO using EDTA-tubes to avoid coagulation and analyzed using routine methods. qPCR analysis on striatum and cortex was performed 24hr after tMCAO as described above.

**Protein-free dietary restriction and functional recovery after stroke (Figure 4)**

The purpose of this experiment was to determine whether a protein-free diet as described above promotes functional recovery after stroke. We used an experimental design with the between-subjects factor of diet (complete diet vs. protein-free DR, n=14/group) and the within-subjects factor of day (2, 7, 14). Rats were maintained on the experimental diets for 6 days prior to and 2 days after tMCAO. Thereafter all rats had *ad libitum* access to complete diet. Behavior was assessed on days 2, 7 and 14 after tMCAO.

**Supplementary References**

1. Koizumi J, Yoshida Y, Nakazawa T, Ooneda G (1986) Experimental studies of ischemic brain edema I: A new experimental model of cerebral embolism in rats in which recirculation can be introduced in the ischemic area. Jpn J Stroke 8: 1-8.

2. Reglodi D, Somogyvari-Vigh A, Maderdrut JL, Vigh S, Arimura A (2000) Postischemic spontaneous hyperthermia and its effects in middle cerebral artery occlusion in the rat. Exp Neurol 163: 399-407.

3. Harvey BK, Richie CT, Hoffer BJ, Airavaara M (2011) Transgenic animal models of neurodegeneration based on human genetic studies. J Neural Transm 118: 27-45.

4. Chen ST, Hsu CY, Hogan EL, Maricq H, Balentine JD (1986) A model of focal ischemic stroke in the rat: reproducible extensive cortical infarction. Stroke 17: 738-743.

5. Airavaara M, Shen H, Kuo CC, Peranen J, Saarma M, et al. (2009) Mesencephalic astrocyte-derived neurotrophic factor reduces ischemic brain injury and promotes behavioral recovery in rats. J Comp Neurol 515: 116-124.

6. Lin TN, Chen, S.T., He, Y.Y., Cheung, W.M. Hsu, C.Y. (2009) Three-Vessel Middle Cerebral Artery Occlusion Model. In: Chen JC, editor. Animal Models of Acute Neurological Injuries. Humana Press, 999 Riverview Drive, Suite 208, Totowa, NJ 07512, USA: Humana Press. pp. 141-153.

7. Bederson JB, Pitts LH, Tsuji M, Nishimura MC, Davis RL, et al. (1986) Rat middle cerebral artery occlusion: evaluation of the model and development of a neurologic examination. Stroke 17: 472-476.

8. Rozen S, Skaletsky H (2000) Primer3 on the WWW for general users and for biologist programmers. Methods in molecular biology 132: 365-386.

9. Kirsch JR, D'Alecy LG (1979) Effect of altered availability of energy-yielding substrates upon survival from hypoxia in mice. Stroke 10: 288-291.

10. Combs DJ, D'Alecy LG (1987) Motor performance in rats exposed to severe forebrain ischemia: effect of fasting and 1,3-butanediol. Stroke 18: 503-511.

11. Rehncrona S, Rosen I, Smith ML (1985) Effect of different degrees of brain ischemia and tissue lactic acidosis on the short-term recovery of neurophysiologic and metabolic variables. Exp Neurol 87: 458-473.

12. Marie C, Bralet AM, Gueldry S, Bralet J (1990) Fasting prior to transient cerebral ischemia reduces delayed neuronal necrosis. Metab Brain Dis 5: 65-75.

13. Go KG, Prenen GH, Korf J (1988) Protective effect of fasting upon cerebral hypoxic-ischemic injury. Metab Brain Dis 3: 257-263.

14. Gietzen DW, Hao S, Anthony TG (2007) Mechanisms of food intake repression in indispensable amino acid deficiency. Annu Rev Nutr 27: 63-78.

15. Hao S, Sharp JW, Ross-Inta CM, McDaniel BJ, Anthony TG, et al. (2005) Uncharged tRNA and sensing of amino acid deficiency in mammalian piriform cortex. Science 307: 1776-1778.

16. Maurin AC, Jousse C, Averous J, Parry L, Bruhat A, et al. (2005) The GCN2 kinase biases feeding behavior to maintain amino acid homeostasis in omnivores. Cell Metab 1: 273-277.
