# Supplementary material for: Short-term Preoperative Dietary Restriction Is Neuroprotective in a Rat Focal Stroke Model
Source: PLoS One. 2014 Apr 4;9(4):e93911. doi: 10.1371/journal.pone.0093911 (PMC3976327; doi:10.1371/journal.pone.0093911)
Supplement: Table S1 — (PDF) [file pone.0093911.s004.pdf]

Supplementary Table 1:
Gene symbols and names

Gene symbol	Gene name
**Neurotrophic and growth factors**	
BDNF	brain-derived neurotrophic factor
CDNF	cerebral dopamine neurotrophic factor
CNTF	ciliary neurotrophic factor
FGF2	fibroblast growth factor 2
GDNF	glial cell line-derived neurotrophic factor
IGF1	insulin-like growth factor 1
MANF	mesencephalic astrocyte-derived neurotrophic factor
NGF	nerve growth factor
NRG1	neuregulin 1
NRTN	neurturin
PSPN	persephin
TGFB1	transforming growth factor, beta 1
**Neurotrophic and growth factor receptors**	
GFRA1	GDNF family receptor, alpha 1
GFRA2	GDNF family receptor, alpha 2
NTRK2/TRKB	neurotrophic tyrosine kinase, receptor, type 2 / tyrosine kinase B
RET	ret proto-oncogene
TGFBR1	transforming growth factor, beta receptor 1
TGFBR2	transforming growth factor, beta receptor 2
TGFBR3	transforming growth factor, beta receptor 3
**Inflammatory response**	
ICAM1	intercellular adhesion molecule 1
TNFA	tumor necrosis factor, alpha
TNFRSF1A	tumor necrosis factor receptor superfamily, member 1A
VCAM1	vascular cell adhesion molecule 1
**Cellular stress response**	
APOE	apolipoprotein E
GPX1	glutathione peroxidase 1
GSTO1	glutathione S-transferase, omega 1
HMOX1	heme oxygenase (decycling) 1
HMOX2	heme oxygenase (decycling) 2
HSPA4/HSP70	heat shock 70 kDa protein 4 / heat shock protein, 70 kDa
HSPA5/GRP78	heat shock 70 kDa protein 5 / glucose-regulated protein, 78 kDa
PTGS2/COX2	prostaglandin-endoperoxide synthase 2 / cyclooxygenase 2
SLC1A3/GLAST	solute carrier family 1 (glial high affinity glutamate transporter), member 3 / glutamate-aspartate transporter
SOD1	superoxide dismutase 1, soluble
SOD2	superoxide dismutase 2, mitochondrial

Gene symbols and names

Gene symbol	Gene name
**Cell death**	
APAF1	apoptotic peptidase activating factor 1
BCL2L11	B-cell CLL/lymphoma 2 (BCL2)-like 11 / bcl-2 interacting protein (BIM)
FADD	Fas (TNFRSF6)-associated via death domain
HIF1A	hypoxia inducible factor 1, alpha subunit
JUN	jun proto-oncogene
RALBP1	ralA binding protein 1
SIRT1	sirtuin 1
TRADD	TNFRSF1A-associated via death domain
XIAP	X-linked inhibitor of apoptosis
**Housekeeping reference gene**	
PPIA	peptidylprolyl isomerase A

Supplementary Table 1:

Primer sequences

Gene symbol	Forward primer	Reverse primer
**Neurotrophic and growth factors**		
BDNF	5'-CGTGGGGAGCTGAGCGTGTG-3'	5'-GCCCCTGCAGCCTTCCTTCG-3'
CDNF	5'-TCGAGGGCTGACTGTGAAGTATGCAA-3'	5'-AGCACAGGCGGTTTTCTTTTCCCT-3'
CNTF	5'-GACCTGACTGCTCTTATGGAATCT-3'	5'-GCCTGGAGGTTCTCTTGGA-3'
FGF2	5'-AAGAGCGACCCACACGTC-3'	5'-CCCTTGATGGACACAACTCC-3'
GDNF	5'-GAGACCGGATCCGAGGTGCC-3'	5'-GAGGAAGTGCCGCCGCTTGT-3'
IGF1	5'-TGACATGCCCAAGACTCAGA-3'	5'-CGTGGCATTTTCTGTTCCTC-3'
MANF	5'-GGCAAAGAAAATCGGTTGTG-3'	5'-CTTCTCCACAGGGATATGGTG-3'
NGF	5'-GGCCACTCTGAGGTGCATAGCG-3'	5'-AGGTGGGTGGAGGCTGGGTG-3'
NRG1	5'-CTCTGCCAACATCACCATTG-3'	5'-TTTCTCCTTCTCCGCACACT-3'
NRTN	5'-TCTGCTCTTGGGTCACCGCCT-3'	5'-AGCAGAGCGCGATACTGGGC-3'
PSPN	5'-AAGCCCCATCAGGGTAACA-3'	5'-CCGGCACAAACCAGGTAA-3'
TGFB1	5'-CCTGGAAAGGGCTCAACAC-3'	5'-CAGTTCTTCTCTGTGGAGCTGA-3'
**Neurotrophic and growth factor receptors**		
GFRA1	5'-GTACTTCGCGCTGCCACT-3'	5'-GCTTTCACACAGTCCAGACG-3'
GFRA2	5'-GTCATCACCACCTGCACATC-3'	5'-AGCACATGCTTAACTCTTTGGA-3'
NTRK2	5'-CGAGGTTGGAACCTAACAGC-3'	5'-CCTTTTCTGGTTTGCAATGAG-3'
RET	5'-CTGGGAGCAGCTCAGCAT-3'	5'-TGTGGACCCCAGGAAGAC-3'
TGFBR1	5'-AAGGCCAAATATTCCCAACA-3'	5'-ATTTTGGCCATCACTCTCAAG-3'
TGFBR2	5'-GAGGACGGCCTGAAATCC-3'	5'-CTCACACACGATCTGGATGC-3'
TGFBR3	5'-TGTGGTACATCTACTCCCACACA-3'	5'-GCGAGGTAGGGACTTGCTG-3'
**Inflammatory response**		
ICAM1	5'-GCAGACCACTGTGCTTTGAG-3'	5'-TCCAGCTCCACTCGCTCT-3'
TNFA	5'-GCCCAGACCCTCACACTC-3'	5'-CCACTCCAGCTGCTCCTCT-3'
TNFRSF1A	5'-AATGAGTGCACCCCTTGC-3'	5'-CCTGGGGGTTTGTGACATT-3'
VCAM1	5'-CAAATGGAGTCTGAACCCAAA-3'	5'-GGTTCTTTCGGAGCAACG-3'
**Cellular stress response**		
APOE	5'-GGATGCCTGGCCGAGGGAGA-3'	5'-ACCGTCAGTTCCTGTGTGACTTGG-3'
GPX1	5'-CGACATCGAACCCGATATAGA-3'	5'-ATGCCTTAGGGGTTGCTAGG-3'
GSTO1	5'-AGGAGCCTGGGGAAGGGAAGC-3'	5'-ACCAGAGACGGCACCTTTGAGA-3'
HMOX1	5'-GTCAAGCACAGGGTGACAGA-3'	5'-CTGCAGCTCCTCAAACAGC-3'
HMOX2	5'-TACGGCACCAGAAAAGGAAA-3'	5'-GTGCTTCCTTGGTCCCTTC-3'
HSPA4	5'-AGCTGAAGAGGGGTCAAGTG-3'	5'-TTTGGAGAAAGGAGCAGCAT-3'
HSPA5	5'-CCGTAACAATCAAGGTCTACGA-3'	5'-AAGGTGACTTCAATCTGGGGTA-3'
PTGS2	5'-CTACACCAGGGCCCTTCC-3'	5'-TCCAGAACTTCTTTTGAATCAGG-3'
SLC1A3	5'-TGGTAGCGGTGATAATGTGG-3'	5'-ATCTTCCCTGCGATCAAGAA-3'
SOD1	5'-CGAGCAGAAGGCAAGCGGTGA-3'	5'-TCGTGGACCACCATAGTACGGC-3'
SOD2	5'-TGGACAAACCTGAGCCCTAA-3'	5'-GACCCAAAGTCACGCTTGATA-3'

Primer sequences

Gene symbol	Forward primer	Reverse primer
**Cell death**		
APAF1	5'-CCTGCTCAGTGGATAAGAAAGTTA-3'	5'-TGAGTGTTCTTCGTAGGTGTGC-3'
BCL2L11	5'-AGATCTGCGCCCAGAGATAC-3'	5'-CTTCCGCCTCTCGGTAATC-3'
FADD	5'-CTACTGCAGCGCCTGGAC-3'	5'-CCCCACATTGTCACACACA-3'
HIF1A	5'-AAGCACTAGACAAAGCTCACCTG-3'	5'-TTGACCATATCGCTGTCCAC-3'
JUN	5'-GGCTGCAGGCGCTGAAGGAA-3'	5'-TCCTCTAGCCGGGCGATCCG-3'
RALBP1	5'-GGCGGCAGGCACTGGTGTAA-3'	5'-GGCAGCGTAGCCTTCAGTCC-3'
SIRT1	5'-TCCTTTCAGAACCACCAAAGCGG-3'	5'-CCGGGAAGTCCACAGCAAGGC-3'
TRADD	5'-CTGGAGGAGAACGAGCTCAC-3'	5'-GAGTCCAGTGGATGGCAAAT-3'
XIAP	5'-GCTTGCAAGAGCTGGATTTT-3'	5'-TGGCTTCCAATCCGTGAG-3'
**Housekeeping reference gene**		
PPIA	5'-TATCTGCACTGCCAAGACTGAGTG-3'	5'-CTTCTTGCTGGTCTTGCCATTCC-3'

Supplementary Table 1:

Fold change normalized to AL 0h

Gene symbol					Sham				tMCAO							
	AL 0h		FA 0h		AL 24h		FA 24h		AL 24h L		AL 24h R		FA 24h L		FA 24h R	
	avg	sem	avg	sem	avg	sem	avg	sem	avg	sem	avg	sem	avg	sem	avg	sem
**Neurotrophic and growth factors**																
BDNF	1	0.448	1.039	0.364	0.500	0.206	0.431	0.161	0.520	0.256	16.82	12.52	6.088	2.698	2.949	2.115
CDNF	1	0.200	0.882	0.126	0.758	0.104	0.797	0.110	0.364	0.087	0.409	0.085	0.829	0.116	0.759	0.091
CNTF	1	0.333	1.002	0.281	0.563	0.062	0.543	0.067	0.781	0.145	1.144	0.279	1.130	0.609	1.283	0.421
FGF2	1	0.101	1.261	0.117	0.986	0.086	1.063	0.077	1.779	0.367	5.687	0.404	1.749	0.328	2.674	0.482
GDNF	1	0.094	0.889	0.070	0.990	0.089	0.927	0.095	1.350	0.243	2.064	0.370	0.885	0.132	0.901	0.127
IGF1	1	0.124	0.949	0.123	0.912	0.088	0.863	0.121	1.029	0.218	1.410	0.358	1.312	0.347	1.452	0.166
MANF	1	0.049	0.939	0.047	1.038	0.061	0.989	0.047	1.156	0.187	2.058	0.173	1.155	0.109	1.473	0.230
NGF	1	0.200	0.673	0.093	0.516	0.120	0.489	0.078	0.692	0.211	0.774	0.151	0.383	0.088	0.867	0.383
NRG1	1	0.140	0.745	0.061	0.724	0.158	0.766	0.145	0.517	0.144	0.337	0.109	0.721	0.119	0.549	0.308
NRTN	1	0.158	0.844	0.078	0.942	0.120	0.940	0.136	1.082	0.039	6.354	1.153	1.116	0.208	2.885	1.121
PSPN	1	0.300	0.564	0.076	0.765	0.082	0.722	0.119	0.868	0.296	0.938	0.310	0.672	0.209	1.027	0.112
TGFB1	1	0.140	0.789	0.059	0.866	0.084	0.788	0.041	0.851	0.029	5.305	0.794	1.326	0.318	1.839	0.581
**Neurotrophic and growth factor receptors**																
GFRA1	1	0.187	0.959	0.097	1.091	0.231	0.849	0.085	1.062	0.151	2.859	0.305	1.438	0.278	1.856	0.365
GFRA2	1	0.143	2.398	1.527	0.685	0.046	1.036	0.279	0.791	0.113	0.843	0.368	1.857	1.196	0.867	0.339
NTRK2	1	0.042	1.215	0.212	1.082	0.101	1.421	0.105	1.623	0.079	1.599	0.379	1.992	0.738	1.250	0.383
RET	1	0.170	1.248	0.239	0.898	0.058	1.096	0.114	1.658	0.298	4.345	0.869	1.841	0.239	3.835	1.161
TGFBR1	1	0.149	0.633	0.081	0.909	0.087	0.816	0.069	0.917	0.136	1.864	0.182	0.998	0.137	1.335	0.257
TGFBR2	1	0.145	0.835	0.097	1.393	0.302	1.250	0.237	1.067	0.092	3.229	0.434	1.361	1.220	2.457	1.155
TGFBR3	1	0.160	1.593	0.186	0.859	0.093	1.334	0.151	1.398	0.187	1.886	0.479	1.296	0.100	1.051	0.413
**Inflammatory response**																
ICAM1	1	0.119	1.117	0.108	1.152	0.129	1.231	0.291	2.023	0.602	14.98	4.182	1.077	0.221	3.114	1.153
TNFA	1	0.148	0.888	0.086	0.799	0.112	0.681	0.081	1.873	1.034	13.12	4.081	0.988	0.343	3.395	1.212
TNFRSF1A	1	0.162	0.714	0.167	0.923	0.140	0.913	0.127	0.926	0.129	3.819	0.559	1.311	0.296	2.416	0.544
VCAM1	1	0.080	0.856	0.055	1.076	0.128	1.218	0.123	1.152	0.097	1.227	0.169	1.394	0.230	1.045	0.129

<u>Fold change normalized to AL 0h</u>

					Sham				tMCAO							
**Gene symbol**	**AL 0h**		**FA 0h**		**AL 24h**		**FA 24h**		**AL 24h L**		**AL 24h R**		**FA 24h L**		**FA 24h R**	
	avg	sem	avg	sem	avg	sem	avg	sem	avg	sem	avg	sem	avg	sem	avg	sem
**Cellular stress response**																
APOE	1	0.184	1.523	0.246	1.284	0.298	1.185	0.212	1.234	0.362	1.300	0.463	1.222	0.301	1.586	0.313
GPX1	1	0.080	1.007	0.131	0.610	0.268	0.150	0.026	0.223	0.125	0.237	0.136	0.252	0.103	0.323	0.084
GSTO1	1	0.161	0.795	0.180	1.288	0.316	0.854	0.234	1.311	0.302	1.593	0.835	1.137	0.370	1.282	0.376
HMOX1	1	0.095	1.061	0.105	1.104	0.098	1.068	0.067	1.507	0.288	65.42	17.53	1.920	0.682	63.45	37.83
HMOX2	1	0.269	0.908	0.089	1.010	0.076	0.788	0.040	1.104	0.111	0.948	0.136	1.146	0.108	0.710	0.072
HSPA4	1	0.181	0.736	0.063	0.662	0.102	0.614	0.048	0.939	0.376	1.247	0.585	1.030	0.290	0.713	0.091
HSPA5	1	0.141	0.809	0.064	0.858	0.041	0.726	0.092	0.976	0.113	1.242	0.153	1.154	0.276	1.206	0.185
PTGS2	1	0.113	1.034	0.085	0.910	0.070	0.750	0.080	1.010	0.135	1.837	0.783	0.996	0.105	1.008	0.204
SLC1A3	1	0.389	0.942	0.119	0.910	0.112	0.781	0.048	0.724	0.110	0.747	0.097	0.979	0.445	1.673	0.385
SOD1	1	0.245	0.591	0.091	1.788	0.280	1.656	0.260	2.813	0.726	3.418	1.134	1.427	0.286	1.888	0.369
SOD2	1	0.105	1.297	0.258	0.866	0.101	1.484	0.390	1.031	0.002	1.633	0.388	1.118	0.051	1.123	0.268
**Cell death**																
APAF1	1	0.112	0.798	0.101	1.015	0.130	0.830	0.057	0.893	0.294	1.211	0.282	0.921	0.113	1.027	0.049
BCL2L11	1	0.200	1.540	0.375	1.277	0.188	0.887	0.104	0.986	0.247	1.632	0.756	1.382	0.295	1.062	0.291
FADD	1	0.119	1.123	0.101	0.937	0.081	0.954	0.062	1.001	0.068	2.000	0.507	1.186	0.215	1.426	0.346
HIF1A	1	0.091	1.298	0.244	1.077	0.166	1.058	0.106	1.100	0.132	1.175	0.152	1.516	0.534	1.228	0.397
JUN	1	0.371	0.783	0.157	0.627	0.084	0.554	0.074	0.681	0.112	3.441	0.682	1.224	0.500	2.134	0.658
RALBP1	1	0.546	0.317	0.124	0.989	0.324	1.038	0.298	1.358	1.199	2.193	1.798	0.485	0.122	0.543	0.231
SIRT1	1	0.111	1.141	0.224	0.925	0.243	0.453	0.023	0.977	0.215	0.799	0.267	0.913	0.072	0.977	0.289
TRADD	1	0.341	0.596	0.044	0.744	0.067	0.469	0.094	0.475	0.060	1.683	0.664	1.773	0.702	0.847	0.198
XIAP	1	0.265	0.878	0.048	0.917	0.107	0.850	0.095	1.258	0.329	0.738	0.127	1.151	0.227	1.467	0.779

Supplementary Table 1:

Fold change relative to PPIA

Gene symbol	AL 0h		FA 0h		Sham AL 24h		Sham FA 24h		tMCAO AL 24h L		tMCAO AL 24h R		tMCAO FA 24h L		tMCAO FA 24h R	
	avg	sem	avg	sem	avg	sem	avg	sem	avg	sem	avg	sem	avg	sem	avg	sem
**Neurotrophic and growth factors**																
BDNF	2.7E-06	1.2E-06	2.8E-06	9.7E-07	1.3E-06	5.5E-07	1.2E-06	4.3E-07	1.4E-06	6.8E-07	4.5E-05	3.3E-05	1.6E-05	7.2E-06	7.9E-06	5.6E-06
CDNF	1.7E-04	3.4E-05	1.5E-04	2.1E-05	1.3E-04	1.8E-05	1.4E-04	1.9E-05	6.2E-05	1.5E-05	7.0E-05	1.5E-05	1.4E-04	2.0E-05	1.3E-04	1.6E-05
CNTF	5.4E-05	1.8E-05	5.5E-05	1.5E-05	3.1E-05	3.4E-06	3.0E-05	3.6E-06	4.3E-05	7.9E-06	6.2E-05	1.5E-05	6.2E-05	3.3E-05	7.0E-05	2.3E-05
FGF2	6.0E-03	6.1E-04	7.6E-03	7.0E-04	5.9E-03	5.2E-04	6.4E-03	4.6E-04	1.1E-02	2.2E-03	3.4E-02	2.4E-03	1.1E-02	2.0E-03	1.6E-02	2.9E-03
GDNF	3.6E-02	3.4E-03	3.2E-02	2.5E-03	3.6E-02	3.2E-03	3.4E-02	3.5E-03	4.9E-02	8.8E-03	7.5E-02	1.3E-02	3.2E-02	4.8E-03	3.3E-02	4.6E-03
IGF1	6.1E-04	7.6E-05	5.8E-04	7.5E-05	5.6E-04	5.4E-05	5.3E-04	7.4E-05	6.3E-04	1.3E-04	8.6E-04	2.2E-04	8.0E-04	2.1E-04	8.8E-04	1.0E-04
MANF	2.9E-02	1.4E-03	2.7E-02	1.3E-03	3.0E-02	1.8E-03	2.8E-02	1.3E-03	3.3E-02	5.3E-03	5.9E-02	4.9E-03	3.3E-02	3.1E-03	4.2E-02	6.6E-03
NGF	9.9E-05	2.0E-05	6.7E-05	9.2E-06	5.1E-05	1.2E-05	4.9E-05	7.8E-06	6.9E-05	2.1E-05	7.7E-05	1.5E-05	3.8E-05	8.7E-06	8.6E-05	3.8E-05
NRG1	1.7E-04	2.4E-05	1.3E-04	1.0E-05	1.2E-04	2.7E-05	1.3E-04	2.5E-05	8.9E-05	2.5E-05	5.8E-05	1.9E-05	1.2E-04	2.0E-05	9.4E-05	5.3E-05
NRTN	1.1E-03	1.8E-04	9.6E-04	8.8E-05	1.1E-03	1.4E-04	1.1E-03	1.5E-04	1.2E-03	4.4E-05	7.2E-03	1.3E-03	1.3E-03	2.4E-04	3.3E-03	1.3E-03
PSPN	1.5E-04	4.5E-05	8.4E-05	1.1E-05	1.1E-04	1.2E-05	1.1E-04	1.8E-05	1.3E-04	4.4E-05	1.4E-04	4.6E-05	1.0E-04	3.1E-05	1.5E-04	1.7E-05
TGFB1	1.0E-01	1.4E-02	8.0E-02	6.0E-03	8.8E-02	8.6E-03	8.0E-02	4.2E-03	8.7E-02	3.0E-03	5.4E-01	8.1E-02	1.4E-01	3.2E-02	1.9E-01	5.9E-02
**Neurotrophic and growth factor receptors**																
GFRA1	4.7E-03	8.8E-04	4.5E-03	4.6E-04	5.2E-03	1.1E-03	4.0E-03	4.0E-04	5.0E-03	7.1E-04	1.4E-02	1.4E-03	6.8E-03	1.3E-03	8.8E-03	1.7E-03
GFRA2	4.2E-04	6.0E-05	1.0E-03	6.4E-04	2.9E-04	1.9E-05	4.4E-04	1.2E-04	3.3E-04	4.7E-05	3.5E-04	1.5E-04	7.8E-04	5.0E-04	3.6E-04	1.4E-04
NTRK2	2.4E-03	9.9E-05	2.9E-03	5.0E-04	2.6E-03	2.4E-04	3.4E-03	2.5E-04	3.9E-03	1.9E-04	3.8E-03	9.0E-04	4.7E-03	1.8E-03	3.0E-03	9.1E-04
RET	1.3E-02	2.1E-03	1.6E-02	3.0E-03	1.1E-02	7.3E-04	1.4E-02	1.4E-03	2.1E-02	3.8E-03	5.5E-02	1.1E-02	2.3E-02	3.0E-03	4.8E-02	1.5E-02
TGFBR1	1.2E-03	1.7E-04	7.4E-04	9.5E-05	1.1E-03	1.0E-04	9.6E-04	8.0E-05	1.1E-03	1.6E-04	2.2E-03	2.1E-04	1.2E-03	1.6E-04	1.6E-03	3.0E-04
TGFBR2	3.2E-03	4.6E-04	2.7E-03	3.1E-04	4.4E-03	9.6E-04	4.0E-03	7.5E-04	3.4E-03	2.9E-04	1.0E-02	1.4E-03	4.3E-03	3.9E-03	7.8E-03	3.7E-03
TGFBR3	4.4E-04	7.0E-05	7.0E-04	8.2E-05	3.8E-04	4.1E-05	5.9E-04	6.6E-05	6.1E-04	8.2E-05	8.3E-04	2.1E-04	5.7E-04	4.4E-05	4.6E-04	1.8E-04
**Inflammatory response**																
ICAM1	1.2E-04	1.5E-05	1.4E-04	1.3E-05	1.4E-04	1.6E-05	1.5E-04	3.6E-05	2.5E-04	7.5E-05	1.9E-03	5.2E-04	1.3E-04	2.8E-05	3.9E-04	1.4E-04
TNFA	3.1E-05	4.6E-06	2.7E-05	2.6E-06	2.5E-05	3.5E-06	2.1E-05	2.5E-06	5.8E-05	3.2E-05	4.1E-04	1.3E-04	3.0E-05	1.1E-05	1.0E-04	3.7E-05
TNFRSF1A	4.5E-03	7.3E-04	3.2E-03	7.6E-04	4.2E-03	6.3E-04	4.1E-03	5.8E-04	4.2E-03	5.9E-04	1.7E-02	2.5E-03	5.9E-03	1.3E-03	1.1E-02	2.5E-03
VCAM1	1.2E+00	9.7E-02	1.0E+00	6.7E-02	1.3E+00	1.5E-01	1.5E+00	1.5E-01	1.4E+00	1.2E-01	1.5E+00	2.0E-01	1.7E+00	2.8E-01	1.3E+00	1.6E-01

Fold change relative to PPIA

Gene symbol	AL 0h		FA 0h		Sham AL 24h		Sham FA 24h		tMCAO AL 24h L		tMCAO AL 24h R		tMCAO FA 24h L		tMCAO FA 24h R	
	avg	sem	avg	sem	avg	sem	avg	sem	avg	sem	avg	sem	avg	sem	avg	sem
**Cellular stress response**																
APOE	4.8E-01	8.9E-02	7.4E-01	1.2E-01	6.2E-01	1.4E-01	5.7E-01	1.0E-01	6.0E-01	1.8E-01	6.3E-01	2.2E-01	5.9E-01	1.5E-01	7.7E-01	1.5E-01
GPX1	3.5E-02	2.8E-03	3.5E-02	4.6E-03	2.1E-02	9.5E-03	5.3E-03	9.2E-04	7.9E-03	4.4E-03	8.4E-03	4.8E-03	8.9E-03	3.6E-03	1.1E-02	3.0E-03
GSTO1	6.6E-05	1.1E-05	5.3E-05	1.2E-05	8.5E-05	2.1E-05	5.7E-05	1.5E-05	8.7E-05	2.0E-05	1.1E-04	5.5E-05	7.5E-05	2.5E-05	8.5E-05	2.5E-05
HMOX1	7.5E-05	7.1E-06	7.9E-05	7.9E-06	8.3E-05	7.3E-06	8.0E-05	5.0E-06	1.1E-04	2.2E-05	4.9E-03	1.3E-03	1.4E-04	5.1E-05	4.8E-03	2.8E-03
HMOX2	3.2E-02	8.7E-03	2.9E-02	2.9E-03	3.3E-02	2.5E-03	2.5E-02	1.3E-03	3.6E-02	3.6E-03	3.1E-02	4.4E-03	3.7E-02	3.5E-03	2.3E-02	2.3E-03
HSPA4	1.3E-02	2.3E-03	9.2E-03	7.9E-04	8.3E-03	1.3E-03	7.7E-03	6.0E-04	1.2E-02	4.7E-03	1.6E-02	7.3E-03	1.3E-02	3.6E-03	8.9E-03	1.1E-03
HSPA5	1.0E-01	1.4E-02	8.3E-02	6.5E-03	8.8E-02	4.2E-03	7.4E-02	9.4E-03	1.0E-01	1.1E-02	1.3E-01	1.6E-02	1.2E-01	2.8E-02	1.2E-01	1.9E-02
PTGS2	5.8E-03	6.6E-04	6.0E-03	5.0E-04	5.3E-03	4.1E-04	4.4E-03	4.7E-04	5.9E-03	7.9E-04	1.1E-02	4.6E-03	5.8E-03	6.1E-04	5.9E-03	1.2E-03
SLC1A3	9.0E-03	3.5E-03	8.4E-03	1.1E-03	8.2E-03	1.0E-03	7.0E-03	4.3E-04	6.5E-03	9.9E-04	6.7E-03	8.7E-04	8.8E-03	4.0E-03	1.5E-02	3.5E-03
SOD1	3.7E-03	9.0E-04	2.2E-03	3.3E-04	6.6E-03	1.0E-03	6.1E-03	9.6E-04	1.0E-02	2.7E-03	1.3E-02	4.2E-03	5.2E-03	1.1E-03	6.9E-03	1.4E-03
SOD2	2.6E-01	2.8E-02	3.4E-01	6.8E-02	2.3E-01	2.6E-02	3.9E-01	1.0E-01	2.7E-01	5.2E-04	4.3E-01	1.0E-01	2.9E-01	1.3E-02	2.9E-01	7.0E-02
**Cell death**																
APAF1	7.6E-04	8.5E-05	6.1E-04	7.7E-05	7.7E-04	9.9E-05	6.3E-04	4.4E-05	6.8E-04	2.2E-04	9.2E-04	2.1E-04	7.0E-04	8.6E-05	7.8E-04	3.7E-05
BCL2L11	7.4E-05	1.5E-05	1.1E-04	2.8E-05	9.5E-05	1.4E-05	6.6E-05	7.8E-06	7.3E-05	1.8E-05	1.2E-04	5.6E-05	1.0E-04	2.2E-05	7.9E-05	2.2E-05
FADD	1.2E-03	1.5E-04	1.4E-03	1.3E-04	1.2E-03	1.0E-04	1.2E-03	7.8E-05	1.2E-03	8.5E-05	2.5E-03	6.3E-04	1.5E-03	2.7E-04	1.8E-03	4.3E-04
HIF1A	1.1E-02	1.0E-03	1.5E-02	2.8E-03	1.2E-02	1.9E-03	1.2E-02	1.2E-03	1.2E-02	1.5E-03	1.3E-02	1.7E-03	1.7E-02	6.0E-03	1.4E-02	4.5E-03
JUN	3.4E-03	1.3E-03	2.7E-03	5.4E-04	2.2E-03	2.9E-04	1.9E-03	2.5E-04	2.3E-03	3.9E-04	1.2E-02	2.3E-03	4.2E-03	1.7E-03	7.3E-03	2.3E-03
RALBP1	1.2E-04	6.4E-05	3.7E-05	1.4E-05	1.2E-04	3.8E-05	1.2E-04	3.5E-05	1.6E-04	1.4E-04	2.6E-04	2.1E-04	5.7E-05	1.4E-05	6.3E-05	2.7E-05
SIRT1	1.0E-04	1.1E-05	1.1E-04	2.3E-05	9.3E-05	2.4E-05	4.5E-05	2.3E-06	9.8E-05	2.2E-05	8.0E-05	2.7E-05	9.2E-05	7.2E-06	9.8E-05	2.9E-05
TRADD	4.8E-04	1.6E-04	2.8E-04	2.1E-05	3.5E-04	3.2E-05	2.2E-04	4.5E-05	2.3E-04	2.9E-05	8.0E-04	3.2E-04	8.5E-04	3.3E-04	4.0E-04	9.4E-05
XIAP	4.0E-04	1.1E-04	3.5E-04	1.9E-05	3.7E-04	4.3E-05	3.4E-04	3.8E-05	5.0E-04	1.3E-04	2.9E-04	5.1E-05	4.6E-04	9.1E-05	5.9E-04	3.1E-04

Supplementary Table 1:

Statistics

Gene symbol	AL 0h vs FA 0h (Student's t-test)	tMCAO AL 24h L vs R	tMCAO AL 24h L vs tMCAO FA 24h L	tMCAO FA 24h L vs R	tMCAO AL 24h R vs tMCAO FA 24h R
**Neurotrophic and growth factors**					
BDNF	0.947	ns	ns	ns	ns
CDNF	0.624	ns	ns	ns	ns
CNTF	0.997	ns	ns	ns	ns
FGF2	0.107	<0.001	ns	ns	<0.001
GDNF	0.354	0.047	ns	ns	0.002
IGF1	0.772	ns	ns	ns	ns
MANF	0.375	0.004	ns	ns	0.038
NGF	0.181	ns	ns	ns	ns
NRG1	0.138	ns	ns	ns	ns
NRTN	0.416	<0.001	ns	ns	0.009
PSPN	0.189	ns	ns	ns	ns
TGFB1	0.209	<0.001	ns	ns	<0.001
**Neurotrophic and growth factor receptors**					
GFRA1	0.855	<0.001	ns	ns	0.026
GFRA2	0.397	ns	ns	ns	ns
NTRK2	0.446	ns	ns	ns	ns
RET	0.409	ns	ns	ns	ns
TGFBR1	0.054	0.004	ns	ns	ns
TGFBR2	0.384	ns	ns	ns	ns
TGFBR3	0.053	ns	ns	ns	ns
**Inflammatory response**					
ICAM1	0.476	<0.05	ns	ns	<0.05
TNFA	0.522	<0.05	ns	ns	<0.05
TNFRSF1A	0.234	<0.001	ns	ns	0.034
VCAM1	0.157	ns	ns	ns	ns

Statistics

Gene symbol	AL 0h vs FA 0h (Student's t-test)	tMCAO AL 24h L vs R	tMCAO AL 24h L vs tMCAO FA 24h L	tMCAO FA 24h L vs R	tMCAO AL 24h R vs tMCAO FA 24h R
**Cellular stress response**					
APOE	0.107	ns	ns	ns	ns
GPX1	0.963	ns	ns	ns	ns
GSTO1	0.407	ns	ns	ns	ns
HMOX1	0.673	0.007	ns	ns	ns
HMOX2	0.792	ns	ns	ns	ns
HSPA4/HSP70	0.198	ns	ns	ns	ns
HSPA5/GRP78	0.259	ns	ns	ns	ns
PTGS2/COX2	0.814	ns	ns	ns	ns

SLC1A3/GLAST	0.890	ns	ns	ns	ns
SOD1	0.161	ns	ns	ns	ns
SOD2	0.347	ns	ns	ns	ns
**Cell death**					
APAF1	0.210	ns	ns	ns	ns
BCL2L11	0.242	ns	ns	ns	ns
FADD	0.441	ns	ns	ns	ns
HIF1A	0.281	ns	ns	ns	ns
JUN	0.621	0.003	ns	ns	ns
RALBP1	0.250	ns	ns	ns	ns
SIRT1	0.589	ns	ns	ns	ns
TRADD	0.227	ns	ns	ns	ns
XIAP	0.661	ns	ns	ns	ns
