# Supplementary material for: Short-term Preoperative Dietary Restriction Is Neuroprotective in a Rat Focal Stroke Model
Source: PLoS One. 2014 Apr 4;9(4):e93911. doi: 10.1371/journal.pone.0093911 (PMC3976327; doi:10.1371/journal.pone.0093911)
Supplement: Table S2 — Analysis of the indicated component from blood plasma from rats fed a complete diet ad libitum (AL) or a protein free (PF) diet for 6 days prior to and 2 days after induction of cortical stroke. (DOCX) [file pone.0093911.s005.docx]

**Supplementary Table S2:** Analysis of the indicated component from blood plasma from rats fed a complete diet *ad libitum* (AL) or a protein free (PF) diet for 6 days prior to and 2 days after induction of cortical stroke.

|  | ALAT  U/L | Albumin  g/L | Protein  g/L | Creatinine  μmol/L | Urea  mmol/L | Glucose  mmol/L | TG  mmol/L | Na  mmol/L | Cl  mmol/L | Prothrombin  mg/dL |
| --- | --- | --- | --- | --- | --- | --- | --- | --- | --- | --- |
| AL (n=11) | 51.64 | 31.36 | 55.70 | 42.70 | 5.00 | 9.15 | 0.64 | 125.78 | 106.63 | 2.43 |
| SEM | 3.85 | 0.28 | 0.76 | 1.15 | 0.35 | 0.23 | 0.07 | 0.77 | 0.39 | 0.29 |
| PF (n=9) | 52.96 | 27.98 | 49.97 | 41.56 | 2.90 | 9.17 | 0.53 | 125.68 | 107.14 | 2.89 |
| SEM | 3.54 | 0.50 | 1.12 | 1.29 | 0.15 | 0.20 | 0.09 | 0.69 | 0.41 | 0.17 |
| T test | 0.81 | <0.001 | <0.001 | 0.51 | <0.001 | 0.96 | 0.37 | 0.93 | 0.38 | 0.20 |
